# Whole-brain, all-optical interrogation of neuronal dynamics underlying gut and vascular interoception in zebrafish

**DOI:** 10.1038/s41467-026-76242-8

**Published:** 2026-08-27

**Authors:** Weiyu Chen, Ben James, Virginie M. S. Ruetten, Sambashiva Banala, Ziqiang Wei, Xueying Yang, Greg Fleishman, Igor Siwanowicz, Mikail Rubinov, Jeremy Delahanty, Mark C. Fishman, Florian Engert, Maneesh Sahani, Luke D. Lavis, James E. Fitzgerald, Misha B. Ahrens

**Affiliations:** 1 Janelia Research Campus, Howard Hughes Medical Institute, Ashburn, VA USA; 2 Department of Neurobiology, Northwestern University, Evanston, IL USA; 3 Gatsby Computational Neuroscience Unit, University College London, London, UK; 4 Department of Biomedical Engineering, Vanderbilt, Nashville, TN USA; 5 Harvard Stem Cell Institute, Harvard University, Cambridge, MA USA; 6 Department of Molecular and Cellular Biology, Harvard University, Cambridge, MA USA; 7 NSF-Simons National Institute for Theory and Mathematics in Biology, Chicago, IL USA

**Keywords:** Neuroscience, Physiology, Neural circuits, Peripheral nervous system

## Abstract

To select behaviors appropriate to their circumstances and needs, animals integrate information about the external environment with information about the state of their bodies, derived from sensing and processing internal signals (interoception). However, the brain-wide circuit activity underlying interoception and its integration with sensorimotor processing remains unclear, partly because of technical barriers to accessing whole-brain activity at the cellular level during physiological perturbations. We developed an all-optical system for whole-brain neuronal imaging in behaving larval zebrafish during optical uncaging of gut- or bloodstream-targeted nutrients and visuo-motor stimulation. Widespread neural activity throughout the brain encoded nutrient delivery, unfolding on multiple timescales across many peripheral and central regions. Evoked activity depended on delivery location and occurred in response to both amino acids and D-glucose, but not L-glucose. Many gut responsive neurons also responded to swimming and visual stimuli, with brainstem areas primarily integrating gut and motor signals and midbrain regions integrating jointly gut, visual, and motor signals. This platform links body-brain communication studies to brain-wide neural computation in awake, behaving vertebrates.

## Abstract (web summary)

Understanding how the brain processes signals from other organs has been limited by a lack of tools. Here the authors introduce an all-optical system for gut or vascular nutrient release during whole-brain imaging in zebrafish.

## Introduction

The importance of interoceptive signals from body organs in information processing in the brain has long been appreciated^1–10^. Interoceptive signals from visceral organs, such as the gastrointestinal tract^11–13^, heart^14^, and lungs^15^, reach the brain through multiple routes, including the vagus nerve^16^, spinal pathways^17^, and circumventricular organs within the brain specialized for detecting chemical changes in the blood^18^. The dorsal vagal complex (DVC, containing the nucleus of the solitary tract [NTS] and other regions) serves as one of multiple hubs in the brain for processing interoceptive information, and connects directly and indirectly to a range of higher-order brain regions (including the parabrachial nucleus [PBN], thalamus, and hypothalamus) for further processing and integration^19,20^, forming a functional network for interoceptive signalling^21–24^.

However, despite rapid progress in our understanding of brain–body interactions in recent years^14,18,23,25–29^, significant challenges remain in capturing the full complexity of interoceptive neural communication. Methods capable of recording whole-brain activity, such as fMRI^30^, often lack the spatial and temporal resolution to monitor single-cell dynamics, while methods capable of recording single-cell dynamics cannot usually record from the whole brain. Whole-brain c-Fos mapping techniques provide a comprehensive overview of brain-wide activation patterns in response to interoceptive signals but do not report temporal dynamics^31–33^. Two-photon calcium imaging and fiber photometry have revealed the organization of sensory responses in subregions of the brain’s interoceptive networks in rodents, but do not allow for the simultaneous monitoring of all interoception-relevant brain regions in a single experiment; furthermore, it is typically challenging to optically access autonomic ganglia of the peripheral nervous system^34–36^. Beyond recording, current methods for manipulating body physiology typically lack spatiotemporal precision in stimulus delivery, can be invasive, can confound chemical and mechanical stimuli, and, in the case of optogenetic delivery, may not mimic natural chemical signaling. For these reasons, the field would benefit from approaches for more subtle internal stimulus delivery and temporally resolved whole-brain recording.

Here, we developed a system that enables access to the dynamics of individual neurons across the entire brain and nodose ganglion during spatiotemporally precise delivery of nutrients to the gut and vasculature in awake, behaving zebrafish. Our all-optical system is minimally invasive and delivers chemical stimulation to the gut or vasculature without causing mechanical deformations or other disruptions of physiology and behavior. We achieved this by delivering chemically caged compounds (such as glucose) via microgavage or microinjection prior to experiments and subsequently photo-uncaging to release the payload compound within the gut or vasculature during whole-brain imaging. To characterize neural dynamics, we developed novel analysis algorithms suited to such multimodal data. To study how interoception integrates with external sensory input and decision-making, we also incorporated visual stimulation and a behavioral readout into the system in the form of fictive swim signals, recorded through electrodes. Together, this enables brain-wide monitoring during controlled delivery of interoceptive signals, in an animal actively engaging with the external environment. Doing so allows for the simultaneous study of interoception, exteroception, and behavior at the whole-brain scale, at single-cell resolution, and without the need for surgery, all in a vertebrate animal. Importantly, the same platform can be readily adapted to study other organ systems, extending its utility beyond the gut-brain axis to broader body–brain interactions. Since much of the central and peripheral nervous systems are conserved across vertebrate species, including mammals, this all-optical technique, combined with the whole-brain analysis methods developed here, promises to reveal fundamental principles of nervous system function that may generally apply across vertebrate brains.

## Results

**Fig. 1:**
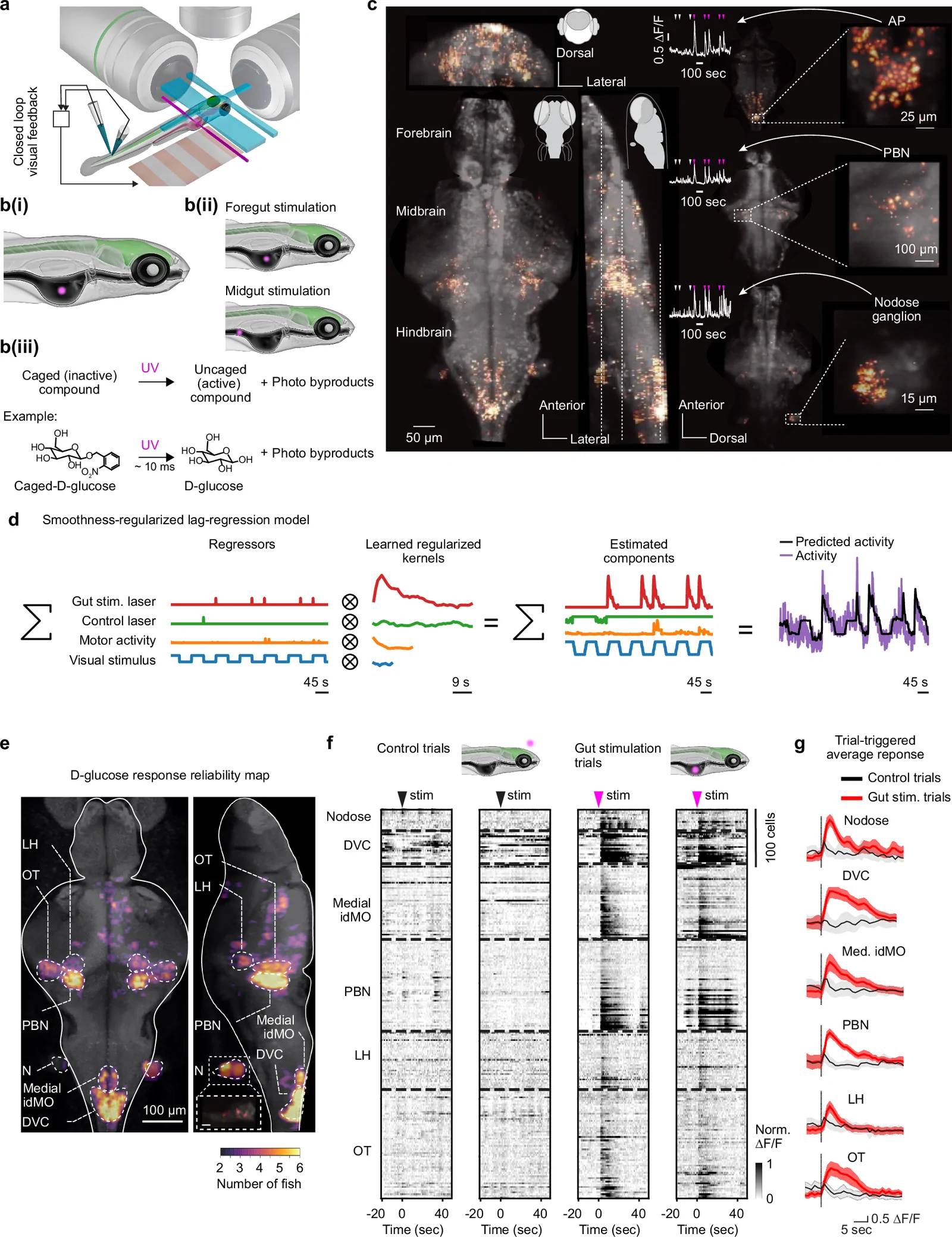
Whole-brain light-sheet imaging with integrated uncaging for all-optical nutrient delivery to the gut. **a** Diagram of the custom light-sheet microscope with integrated optical uncaging. Blue planes denote the excitation light sheets, consisting of swept light pencils that are blocked by an electronic shutter when pointed at the fish’s eyes to prevent direct retinal exposure. Purple beam represents UV illumination for uncaging. The central nervous system of a larval *Tg(elavl3:H2B-jGCaMP7f)* zebrafish is depicted in green. **b** Optical uncaging in the larval zebrafish gut. **b-i**, **ii** Localized nutrient release in the foregut and midgut; purple dot indicates UV beam. **b-iii** Uncaging reaction yields free chemicals within ~10 ms. **c** Example of brain-wide average ∆*F*/*F* over 5 trials during glucose uncaging. Insets show activity traces and images from the area postrema (AP), parabrachial nucleus (PBN), and nodose ganglion. Red arrowheads mark stimulus delivery times. **d** Smoothness-regularized distributed-lag regression model decomposing neuronal activity (purple) into components based on stimuli and motor regressors. Kernels (middle) are regularized for amplitude and smoothness. **e** Average brain-wide activity map in response to glucose uncaging in the gut (*N* = 6 fish). Color indicates the number of fish with significant activation. N: nodose, DVC: dorsal vagal complex, PBN: parabrachial nucleus, LH: lateral hypothalamus, OT: optic tectum, medial idMO: medial inferior dorsal medulla oblongata. Inset is a zoomed-in view of the nodose ganglion. Scale bar is 100 µm. **f** Example raster plots of neuronal activity during control (black arrows) and gut-targeted (red arrows) UV stimulation. Each column represents a single trial. Each row represents one neuron. Time = 0 s marks UV onset. **g** Example average ΔF/F calcium traces (mean ± SEM) from control (gray) versus gut-targeted UV nutrient uncaging trials (red) in representative brain areas.

We extended a whole-brain light-sheet imaging system^37^ to allow for optical uncaging of chemicals in the body of larval zebrafish (Fig. 1a and Supplementary Fig. 1). Optical uncaging was achieved using a steerable ultraviolet (UV) beam (~16 µm^2^ spot area [~4.5 µm FWHM diameter], 355 nm wavelength, Supplementary Fig. 2) that was routed through one of the horizontal illumination objectives and could be steered with a pair of galvo mirrors installed in the light-sheet microscope setup. The setup also contained recording electrodes to monitor fictive-swimming, a visual display under the fish, and optional closed-loop control over the visual stimulus by behavior to provide a virtual reality environment for the fish^37,38^. The setup can easily be used for non-fictive experiments by adding a lens and a camera underneath the imaging chamber^38^ to record tail and fin motion. With this system, we first performed calcium imaging during gut-targeted nutrient release (Fig. 1b-i, ii) in transgenic *Tg(elavl3:H2B-jGCaMP7f)*^39^ larval zebrafish that express jGCaMP7f in almost all neurons, enabling precise uncaging and high-resolution imaging of the entire brain at one to three volumes per second. Prior to experimentation, we introduced one of several caged compounds^40–42^ (Fig. 1b-iii) into the zebrafish gut via microgavage^43^ (Supplementary Fig. 3a and “Methods”). The transparency of larval zebrafish allowed us to precisely uncage caged nutrients already present in the gut using UV light.

This optical method bypassed sensory cues typically associated with nutrients, such as taste, both during experimental trials and sample preparation, enabling us to maintain accurate spatial and temporal control of nutrient delivery during whole-brain imaging. It also minimized imaging artifacts or behavioral disruptions commonly associated with mechanical nutrient delivery methods (Supplementary Fig. 3b, c). To disambiguate neural encoding of gut-interoceptive signals from visual responses that might be elicited by scattered UV light, we also targeted the beam to regions outside the gut (either outside the fish, on the tail, or on the swim bladder) during control trials.

Our palette of caged compounds (Fig. 1b-iii) allowed us to measure neural responses to physiologically meaningful nutrients. In the wild, the larval zebrafish diet^44^ includes paramecia^45^, which contain amino acids including glutamate, and carbohydrates that are broken down into glucose during digestion (and the adult diet contains further carbohydrate-rich organisms^46,47^), making both glutamate and glucose relevant nutrients for zebrafish. Furthermore, glucose is already known to activate enteroendocrine cells (EECs) in the zebrafish intestine^2^, supporting its physiological relevance as a stimulus in studies of gut-brain signaling.

Uncaging of caged glucose^41^ elicited strong, time-locked responses across multiple regions of the peripheral nervous system and brain, including the area postrema (AP), parabrachial nucleus (PBN), and nodose ganglion (Fig. 1c and Supplementary Video 1), without eliciting adverse behavioral reactions (Supplementary Fig. 3d–f). To quantify neuronal responses to gut-lumen nutrient uncaging and assess their consistency across animals, we developed a regression model that predicts single-neuron activity based on time series of chemical stimulus release in the gut, visual input, and behavior (Fig. 1d and “Methods”). Rather than a priori defining a single kernel shape for all cells globally, we used lagged regressors to simultaneously estimate linear response kernels for each regressor modality and each individual cell independently. This allows for differences in response kinetics both between cells and between regressors, thereby improving predictive accuracy by automatically tuning to each cell’s response profile. Importantly, the kernels were constructed in a fashion allowing for tractable closed-form solutions (“Methods”), enabling efficient computation even with hundreds of thousands of cell time series and hours of recording time. This method can quickly estimate and decompose the contributions of gut-chemo-sensory stimuli, control UV pulses, visual stimuli, and motor signals to the activity of every neuron recorded throughout the brain.

By recording from a cohort of fish and applying our analysis methods, we created a whole-brain map (“Methods”) of encoding of gut-chemo-sensory interoception showing the anatomical location of cells identified as glucose-sensitive (Fig. 1e), which can be decomposed into individual activity traces (Fig. 1f) and regional averages (Fig. 1g). We identified many brain areas that responded consistently across fish, including the AP (6/6 fish), PBN (6/6 fish), dorsal vagal complex (DVC; 6/6 fish), a dorsomedial hindbrain cluster within the inferior dorsal medulla oblongata (medial idMO; 6/6 fish), optic tectum (OT; 5/6 fish), lateral hypothalamus (LH; 4/6 fish), and nodose ganglion (4/6 fish). The lower incidence of activation in OT, LH, and nodose across animals likely reflects differences in activation patterns across animals or increased registration error due to reduced imaging quality at greater depths, rather than a total lack of neural activation in those regions. Thus, the methodology identifies a vertebrate whole-brain map of neuronal dynamics underlying gut interoception at single-cell resolution. Notably, our whole-brain imaging system allows for all these regions and their dynamics to be efficiently and reliably identified in a single experiment (Fig. 1c), rather than requiring multiple experiments focused on single brain areas.

**Fig. 2:**
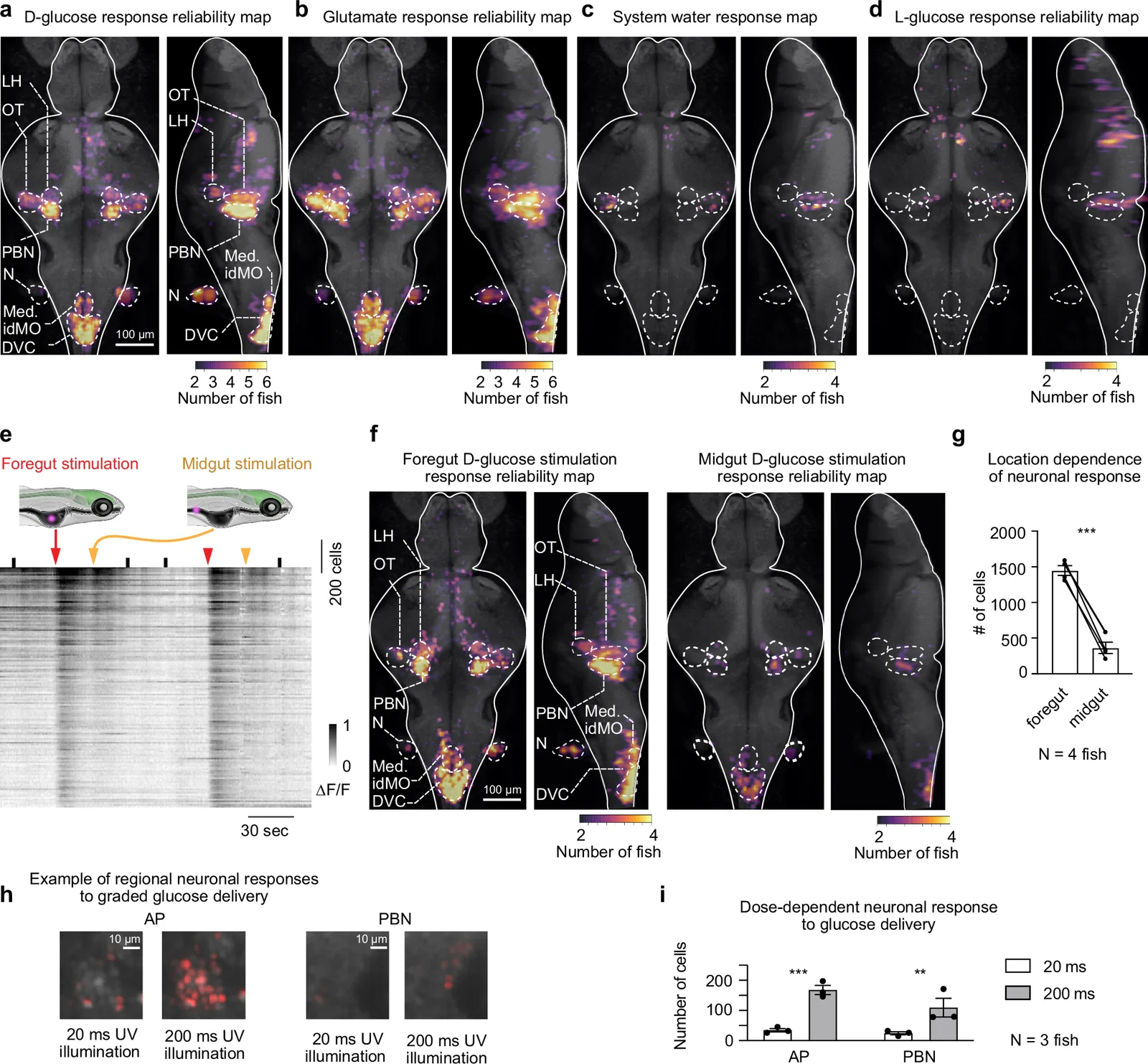
Chemical and spatial topography of neuronal responses to nutrients. **a** Average brain-wide activity map in response to glucose uncaging in the gut (*N* = 6 fish). Color indicates the number of fish with significant activation per region. Same as Fig. 1e. Repeated here for easier comparison with (**b**, **c**). **b** Average brain-wide activity map in response to glutamate uncaging in the gut (*N* = 6 fish). **c** Control experiment showing the average brain-wide activity in response to UV stimulation with only fish water in the gut (*N* = fish). **d** Control experiment showing the average activity during UV uncaging of L-glucose in the gut (*N* = 4 fish). **e** Example raster plot of neuronal activity during foregut and midgut nutrient uncaging. Diagrams show UV location (dots) on the foregut (red arrowhead) and midgut (yellow arrowheads). Each row represents a single neuron. Black tick marks the onset of control UV stimulation, where UV was targeted outside of the fish. **f** Average brain maps of foregut-responsive (left) versus midgut-responsive (right) neurons. **g** Number of responsive neurons for foregut- versus midgut-nutrient uncaging (*N* = 4 fish). Bar graph represents mean ± SEM. Statistical significance was assessed with a two-tailed paired t-test (*t*(3) = 15.75, *p* = 0.0006, 95% CI [−1306, −867], *r*^2^ = 0.988). **h** Example activation patterns in AP and PBN during 20 and 150 ms irradiation. **i** Number of responsive neurons in AP and PBN during 20 and 200 ms irradiation (*N* = 3 fish). The bar graph represents mean ± SEM. Statistical significance was determined using multiple two-tailed unpaired t-tests. (AP group: *t* = 5.358, d*f* = 8, *p* = 0.000679; PBN group: *t* = 3.416, d*f* = 8, *p* = 0.009144).

The platform can be used to study whole-brain neuronal representations of a variety of nutrients in the digestive system by taking advantage of advances in chemistry to cage a wide variety of compounds^40–42,48–51^. We thus compared neural responses to caged glucose (Figs. 1e and 2a) and caged glutamate (Fig. 2b). Although fine-scale differences were apparent, the brain areas responding to glutamate uncaging in the gut overlapped heavily with those responding to glucose, suggesting that population codes within these brain regions encode chemical identity but that there is no region-level specialization between the two nutrients. To test how well the set of identified cells can decode chemical identity, we performed a cross-validated linear discriminant analysis using the three-dimensional spatial coordinates of selected cells for each experimental condition. The classifier correctly identified the delivered compound ~66% of the time, a value above chance but reflective of the high degree of overlap between neurons activated in response to glucose or glutamate release.

We performed additional control experiments to rule out potential UV-light artifacts on brain responses. We first replaced the caged compound solution with fish tank water and found a negligible number of responding cells (Fig. 2c, responses in 0/6 fish for every glucose-sensitive brain region). We next verified that the chemical cages released by uncaging were not detected by the gut by replacing caged D-glucose (so far designated caged glucose) with caged L-glucose (Supplementary Fig. 4a–i), a metabolically inactive enantiomer. Uncaging L-glucose did not activate the brain regions activated during D-glucose or glutamate stimulation (Fig. 2d, responses in 0/6 fish), showing both that the gut selectively senses D-glucose, and not L-glucose, and that the chemical cages elicit little or no response. These controls validate the accuracy and specificity of the paradigm. Notably, multiple compounds can be introduced to the same fish consecutively (Supplementary Fig. 4j), in principle enabling the creation of a cell-by-cell map across the entire brain to define the gut-chemosensory receptive field of individual neurons.

Thanks to having optical access to the entire body, the system can be used to probe the brain’s representation of nutrient presence at specific locations within the gastrointestinal tract. We photo-uncaged glucose in the foregut and midgut of the same fish and observed that uncaging in the foregut elicited strong brain-wide responses, while uncaging in the midgut elicited substantially weaker responses (Fig. 2e–g). This effect likely arises both from the higher density of mucosal afferent endings specialized for detecting chemical stimuli in proximal versus distal intestine^52–54^, and from greater vagal innervation of the foregut relative to midgut at this developmental stage^1^. Thus, all-optical studies of interoception can be used to probe the topographic relationship between the location within organs and brain activity, showing that the brain encodes foregut content more reliably than midgut content.

Our UV uncaging approach enabled control over the amount of glucose released, allowing for the examination of how brain activity varies with gut glucose concentration. To avoid tissue damage, we varied the duration, rather than the intensity, of UV irradiation. Longer uncaging durations, and thus higher glucose concentrations, led to a greater number of activated neurons in the AP and PBN (Fig. 2h, i), demonstrating that our optical uncaging approach enables dose-dependent control of glucose release and corresponding neural activation.

In natural settings, the brain does not process internal signals in isolation. Rather, it combines information about the body’s nutrient state with external sensory cues to dynamically shape behavior^55–61^. To understand how the brain integrates interoceptive information with exteroceptive and motor signals, we next examined the interaction between gut-chemo-sensory stimulation, visual processing, and behavior. Here, we presented drifting grating stimuli to the zebrafish to elicit optomotor responses while recording both motor activity and brain responses. We chose the optomotor response (OMR) paradigm because it involves sustained locomotion driven by visual motion cues and thus directly relates to energy expenditure. Previous research has shown that larval zebrafish adapt their swimming behavior by entering a passive state when faced with ineffective swims (swim bouts eliciting no visual feedback)^62^, which lessens metabolic costs. We thus reasoned that gut-derived signals reflecting internal energy status could influence the OMR by modulating how visual and motor information are integrated. However, following gut-lumen nutrient uncaging, zebrafish continued to display optomotor responses, and visual and motor maps created during gut-nutrient uncaging experiments resembled previous findings^63,64^ (Supplementary Fig. 5), indicating that our paradigm does not override visuo-motor processing and behavior.

**Fig. 3:**
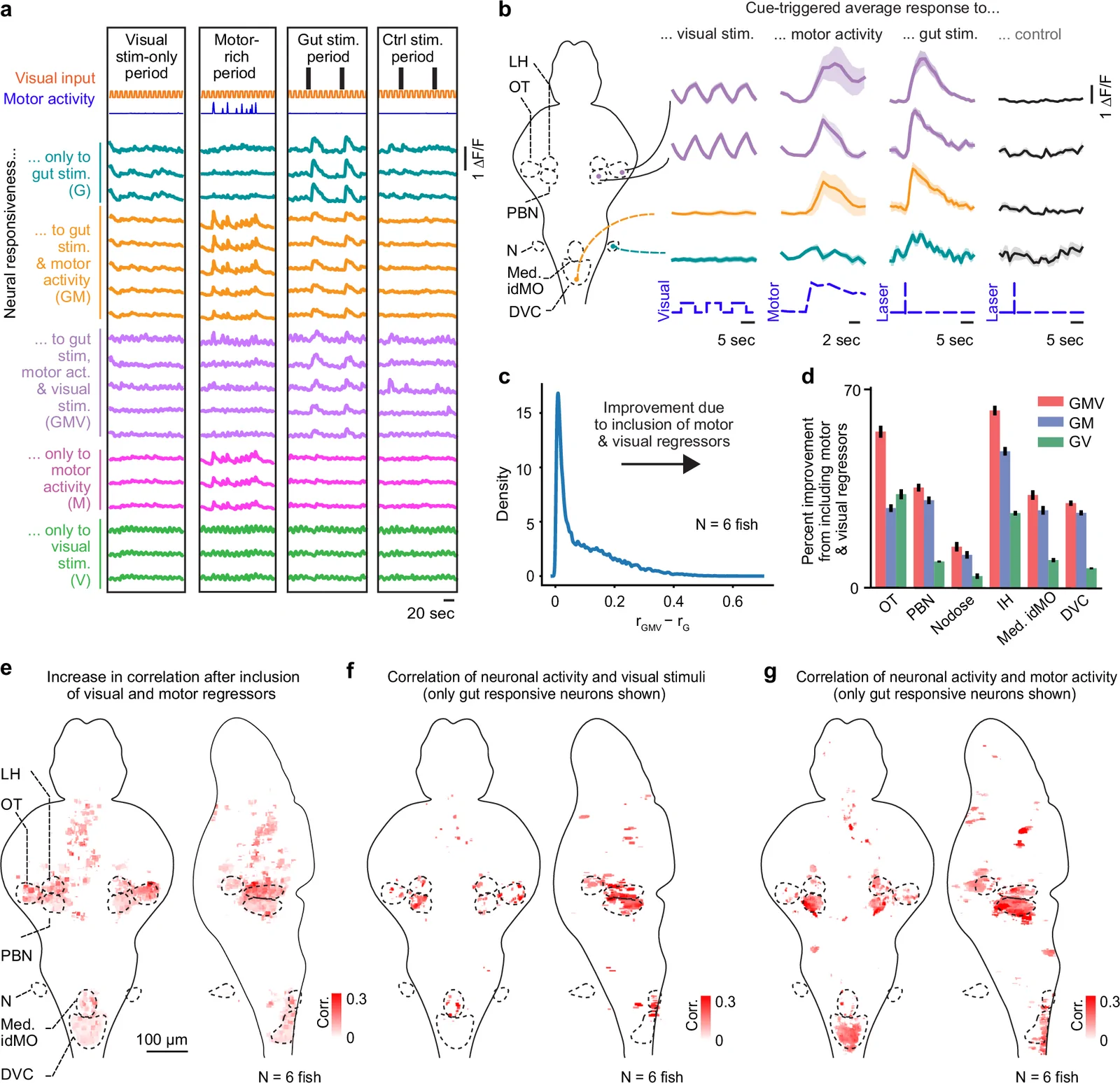
Integration of sensory, motor, and gut-chemo-sensory signals across the brain. **a** Example calcium traces from individual gut-chemo-responsive neurons during four distinct experimental periods (visual, motor, control, and gut). Each row represents a single neuron. The top orange trace indicates visual motion, with upward segments representing forward movement of the visual gratings and downward segments indicating no motion. The blue trace reflects motor power derived from a sliding window of the tail voltage signal. The red arrowheads mark the onset of each UV stimulation on the foregut. The black arrowheads mark the onset of control UV stimulation just outside the fish. **b** Example average ∆*F*/*F* responses of representative single neurons from major gut-chemo-responsive brain regions. Each dot on the schematic (left) marks the location of a sampled neuron. Plotted are the median and SEM. The dashed lines at the bottom mark the timing of visual, motor, gut, and control stimulation. **c** Distribution of ${r}_{{GMV}}-{r}_{G}$, the difference in correlation between a neuron’s activity and a linear model incorporating gut, motor, and visual information and the correlation between the neuron and a linear model only incorporating gut information. Note the strong right tail, indicating the presence of multimodal neurons. **d** Bar plots of percentage change in correlation from the gut model and a model including gut, motor, and visual information (red), a model including gut and motor information (blue), and a model including gut and visual information (green), separated by brain region. Note that regions found in the hindbrain have minimal improvement when including visual information. G, M, V stands for gut, motor, and visual, so that, for instance, GMV represents a model based on gut, motor, and visual signals, and GM represents a model based on gut and motor signals. *N* = 6 fish with *n* = 825, 1630, 218, 850, 617, and 1611 for regions OT, PBN, nodose, LH, medial idMO, and DVC, respectively. The bar graph represents mean ± SEM. **e** Spatial map of ${r}_{{GMV}}-{r}_{G}$ across the brain (*N* = 6 fish). Increased color saturation indicates additional predictability gained when incorporating motor and visual information in models of neural activity. **f** Spatial map indicating the correlation of gut-responsive neurons with the visual signal across the brain (*N* = 6 fish). The color scale indicates the correlation coefficient between neuronal activity and visual motion. (See “Methods” for analysis details). **g** Spatial map of the same gut-responsive neurons showing correlations with the motor signal (*N* = 6 fish). Color indicates the correlation coefficient between neuronal activity and motor power. (See “Methods” for analysis details).

To assess interactions between the stimulus modalities at the neuronal level, we investigated whether and to what extent neurons that encode gut-chemo-sensory stimuli also encode visual information and motor state. To do so, we assessed the predictive benefits of linear models incorporating all regressor modalities (gut-chemo-sensory stimulation, control UV stimulation, visual stimulation, and motor activity) over alternative models containing only gut-chemo-sensory stimulation and control UV regressors (Fig. 1d and “Methods”). A subset of gut-chemo-sensitive neurons responded to both gut-nutrient uncaging and motor activity, and another responded to a combination of gut-nutrient uncaging, motor activity, and visual stimulation (Fig. 3a, b). In total, 26% of cells across six fish showed improvements in the correlation between real and predicted responses of 0.1 or greater when visual and motor information was included (Fig. 3c). We attempted to further identify which modalities most strongly affect responses in gut-chemo-sensitive neurons (Fig. 3d) and the spatial patterns of multimodal neurons (Fig. 3e) by computing the average increase in performance when incorporating either motor activity (GM, incorporating gut-nutrient and motor signals), visual stimulation (GV, incorporating gut-nutrient and visual signals), or both (GMV, incorporating all three signals) as a function of brain area. We also computed raw correlations between neural activity and visual stimulation (Fig. 3f) and motor activity (Fig. 3g). Throughout the brain, many gut-chemo-sensitive neurons also exhibited motor and visual responses, with gut-chemo-sensitive areas within the posterior brainstem responsive to motor activity, and gut-chemo-sensitive areas within the midbrain responsive to both motor activity and visual stimulation.

**Fig. 4:**
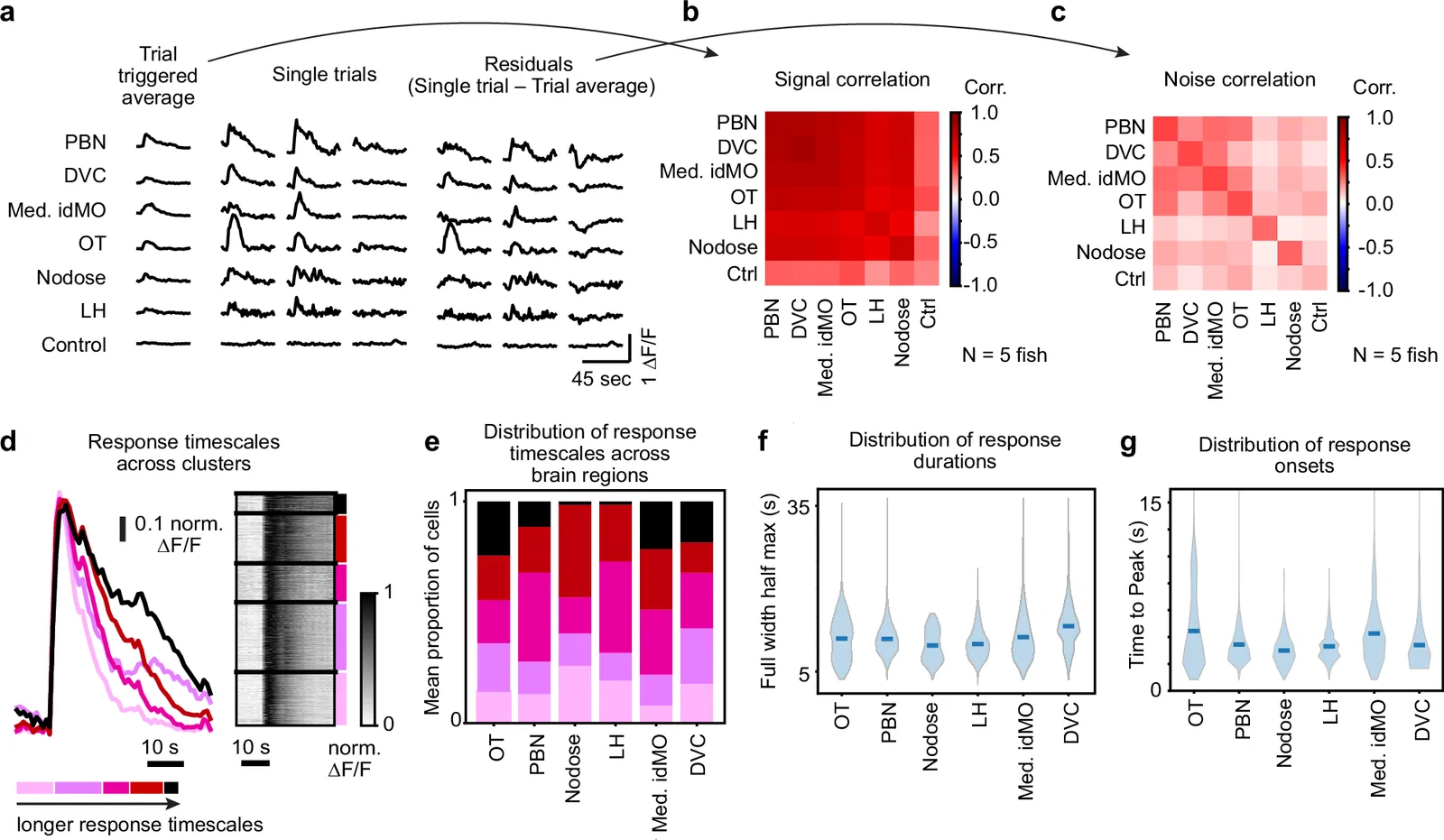
Correlations and brain-wide dynamics of gut-responsive neurons. **a** Example average (left), trial responses (middle), and residuals (right) for example gut-responsive neurons in identified areas as well as control. Each trace is approximately 55 s long. **b** Mean signal correlation between gut-responsive neurons in identified regions. Note the strong signal correlations present between brain regions, indicating similar representations of gut chemosensory stimulation across the brain (*N* = 5 fish). **c** Mean noise correlation. Note that areas within the brain show high noise correlations between one another, as opposed to nodose or control (*N* = 5 fish). **d** Results of clustering average responses to gut-nutrient uncaging for all gut-responsive neurons identified in a single fish. *Left:* mean responses within each cluster demonstrate the variability in decay time found across the brain. *Right:* raster plot of all responses grouped by cluster (as in left). **e** Mean proportion of cells found in each cluster for identified gut-responsive areas. Clusters are colored by ordering decay timescales, as in (**d**). Note that all areas contain all different response types with varying frequency. **f** Distribution of full-width half-maxes in each region. **g** Distribution of times-to-peak in each region.

As our system images the whole brain nearly simultaneously, we have the additional benefit of being able to observe trial-to-trial correlations between gut-chemo-responsive neurons in disparate regions (Fig. 4a and see Supplementary Fig. 6a for additional traces and Supplementary Fig. 7 for alternate correlation measures). As expected, we found that the signal correlation^65^ between gut-chemo-responsive neurons (the correlation between trial-average gut responses across identified cells, “Methods”) was high across gut-chemo-responsive brain areas, which included anatomically distant regions (Fig. 4b). Noise correlations (correlations in trial-to-trial variations between cells, “Methods”) are commonly used in neuroscience to approximate the degree of functional connectivity between pairs of neurons^66^ (correlated fluctuations present in two neurons could arise from spontaneous events in one neuron impacting the other, from spontaneous events occurring in common inputs to both neurons, or trial-to-trial variability in the stimulus due to e.g., gradual depletion of the caged compound in the gut lumen). We quantified average noise correlations within pairs of neurons across brain regions, observing heightened values between cells within DVC, parabrachial nucleus, and the medial idMO compared to control populations (cells in the nodose ganglia that did not respond to the nutrients delivered to the gut lumen) (Fig. 4c). Perhaps surprisingly, gut responsive neurons in the nodose ganglia and LH showed negligible noise correlations, arguing against the source being stimulus variability. This can be understood within the broader context of connectivity: the nodose ganglia and LH receive interoceptive signals through only partially overlapping pathways, with the nodose containing primary visceral sensory neurons^67^, whereas LH interoceptive access is largely mediated by multisynaptic pathways and blood-borne signals sensed through circumventricular interfaces, such as the median eminence^68,69^. These quantifications are consistent with a tightly coupled functional network for interoceptive signaling across the brain^21–24^.

We next inspected the response time courses across the brain. While the responses of most neurons showed an onset time of 5 s or less, their decay times varied from ~10 s to over a minute. We used k-means clustering to divide the average responses for each fish into five clusters, resulting in clusters segregated largely on the basis of decay time (Fig. 4d). By performing this analysis on each fish individually and then sorting the clusters by decay time, we could compare how decay times vary across the fish brain. We initially hypothesized that these varying time courses would be due to region-specific properties, with peripheral regions like the nodose exhibiting faster responses and downstream regions like the PBN exhibiting more integrative and sustained responses. We instead found that all regions contained neurons tiling most of the temporal spectrum, with each brain region containing multiple different response types (Fig. 4e). Despite the broad distributions of timescales (Fig. 4f and Supplementary Fig. 7b) and response latencies (Fig. 4g) contained within each group, some brain regions nonetheless showed statistically significant differences (Supplementary Fig. 6b). Notably, the nodose ganglia had a significantly shorter response than most other detected brain regions, consistent with this being a peripheral sensory locus of the vagal interoceptive nervous system. While it is possible that this is a readout of the lingering uncaged chemical in the gut, it may indicate, consistent with the above analysis of inter-region correlation, that these intermingled timescales are due to across-area communication.

**Fig. 5:**
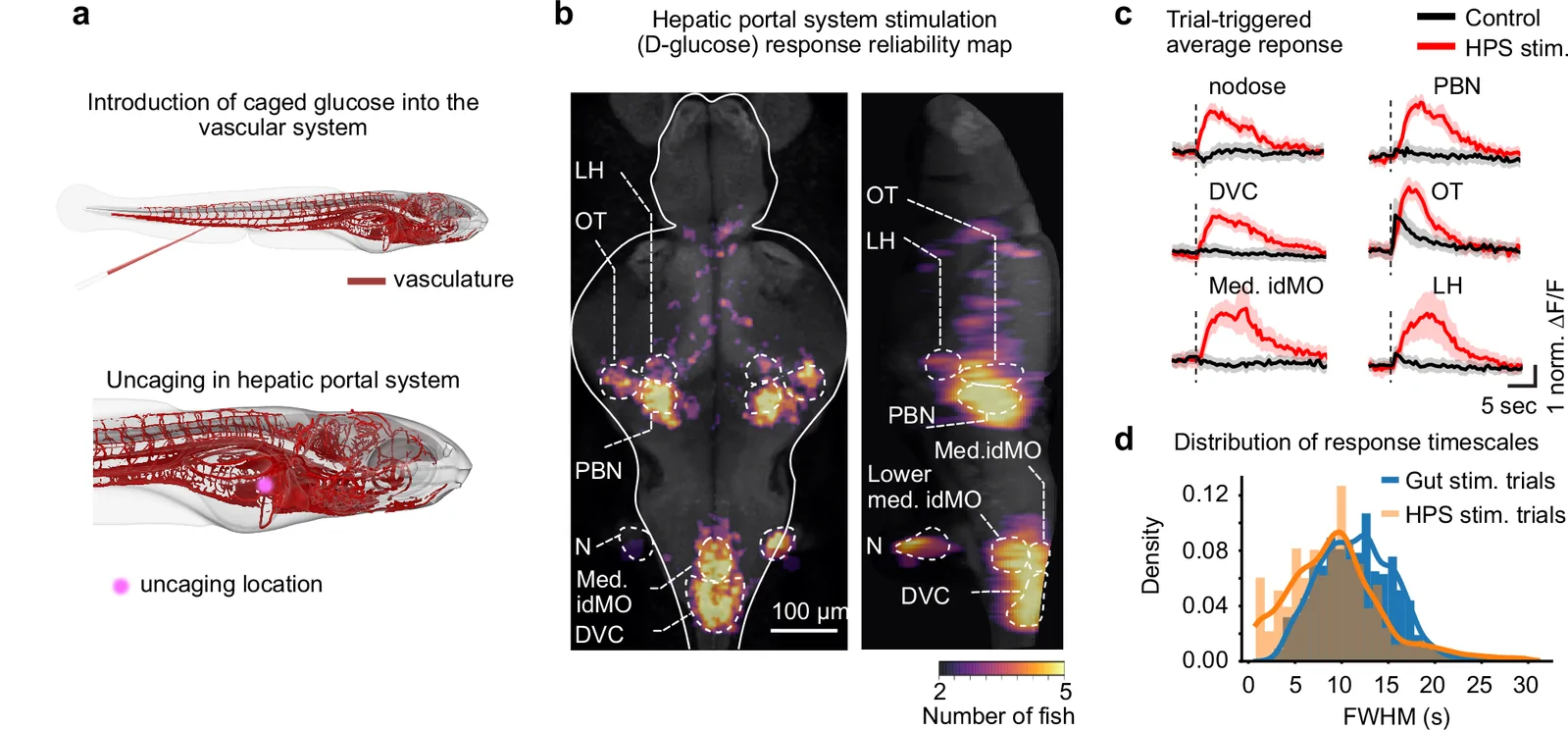
Evoked neuronal activity from uncaging in the circulatory system. **a** Schematic showing the introduction of caged glucose into the circulation via tail vein injection prior to the experiment (top) and uncaging location targeting the hepatic portal system (magenta spot, bottom). **b** Average brain-wide activity map in response to glucose uncaging in the portal vein (*N* = 5 fish). Color indicates the number of fish in which each region showed significant activation. N: nodose, DVC: dorsal vagal complex, PBN: parabrachial nucleus, LH: lateral hypothalamus, OT: optic tectum, medial idMO: medial inferior dorsal medulla oblongata, ventro-medial idMO: ventro-medial inferior dorsal medulla oblongata. The scale bar is 100 µm. **c** Example average Δ*F*/*F* calcium traces (mean ± SEM) from control (gray) versus HPS-targeted UV trials (red) in representative brain areas. **d** Distribution of FWHMs for responses evoked from gut uncaging (blue) and HPS uncaging (orange). Note the longer responses to gut-nutrient uncaging.

Following absorption across the gastrointestinal epithelium, glucose enters capillaries that drain into the venous tributaries of the hepatic portal vein [HPV]^70^. This pathway delivers nutrient-rich blood directly to the hepatic sinusoids, collectively forming the hepatic portal system [HPS]. This first-pass arrangement allows the liver to buffer postprandial rises in systemic blood glucose through dynamic glycogen storage and mobilization^71^. Previous work has shown that both the HPV and the gastrointestinal tract of vertebrates are innervated by glucose-responsive nerve fibers that transmit this information to the brain^70,72^; however, it is not known whether the brain differentially encodes the presence of glucose within the gut lumen versus the hepatic portal system. To address this question, we injected caged glucose into the tail vein and allowed it to circulate and distribute throughout the bloodstream (Fig. 5a and “Methods”). We then directed UV light to the vascular bed draining the gut near the liver, uncaging the compound predominantly within the HPS. Glucose was released in the HPS-activated cells within the DVC, medial idMO, PBN, LH, OT, and nodose ganglion (Fig. 5b, c), the same regions previously identified as responsive to glucose uncaging in the gut lumen. In addition, we identified a region within the ventro-medial inferior dorsal medulla oblongata [ventro-medial idMO] that was selectively activated during glucose release in the HPS but not during glucose release within the gut lumen. Notably, the response durations, quantified by the full width at half maximum (FWHM), were substantially shorter for HPS uncaging than gut lumen uncaging (Fig. 5d, Mann–Whitney *U*, *p* < 0.001). This may reflect more rapid clearance of portal glucose, which is continuously washed out by blood flow, relative to luminal glucose, which can linger at the epithelium. Alternatively, it may reflect a multi-phase response in which glucose is first sensed at the gut epithelium and then within the portal vein following absorption. Together, these findings demonstrate that our approach enables brain-wide interrogation of how glucose signals are detected at different points along the path from the gut to systemic circulation.

In sum, we developed a system for whole-brain interrogation of nutrient sensation in the gut and circulatory system of awake and behaving larval zebrafish. This presents a paradigm for merging analyses of interoceptive and exteroceptive processing, as the system allows for holistically combining both stimulation modalities as well as behavior into a single framework. This work enables the investigation of the neural basis of complex behaviors that rely on interoceptive and exteroceptive signals, unifying systems neuroscience with the study of brain-body communication.

## Discussion

The system developed in this work was applied primarily to the study of gut-brain communication. However, as a general paradigm for whole-brain imaging during chemical manipulation of bodily tissue, it is not restricted to the gut. As proof of principle beyond luminal gut-nutrient uncaging, we also applied this approach to the circulatory system by uncaging glucose directly within the portal vein and simultaneously recording brain-wide neural activity. The vast array of existing caged chemicals^40–42,48–51^ implies that the same paradigm can be used for studies of the brain and behavioral response to many other important perturbations of body physiology. By systematically varying the caged compounds within the gut, future studies will dissect how the brain encodes specific nutrient classes, detects harmful substances, and integrates metabolic signals into feeding and homeostatic regulation. By making use of the zebrafish’s ability to absorb many small molecules through the bath or injecting caged compounds directly into the bloodstream or specific tissues, applications beyond the gut and circulatory system include the study of neural encoding of light-induced liver acidification^73–76^, hormones in the bloodstream^77,78^, optogenetically induced apoptosis in various body organs^79^, and more. These future applications promise insights into the brain’s encoding of many body-wide physiological signals, as well as their influence on perception, memory, and behavior, that were hitherto inaccessible.

The ability to monitor such body–brain interactions at the whole-brain level at single-cell resolution enables precise dissection of the mechanisms by which internal physiological states modulate perception and action. In this work, by incorporating a body-wide uncaging module within a virtual reality and whole-brain imaging setup, we provided a noninvasive way to modulate body physiology while simultaneously tracking behavior and imaging neural responses across the entire brain, allowing us to examine how gut-derived signals influence ongoing motor and sensory processing. The influence of bodily state on brain-wide processing is extensive, as exemplified by the impact of sickness or hunger on behavior, emotional state, and as reinforcement signals in learning^80–82^. Interactions between these states and cognitive processes are relatively understudied, in part because accessing the brain and body simultaneously has been challenging, and in part because the sites of interaction are often unknown. Whole-brain imaging during body manipulation overcomes these challenges, allowing for the monitoring of interactions across brain areas, implementing interoceptive and exteroceptive computations. Beyond the virtual reality environments used here, it would be interesting to study other scenarios, including the influences of body physiology on perception and action in prey capture^80,81^ and learning^83,84^. Eventually, the merging of systems neuroscience with studies of interoception promises to lead to an understanding of the body-wide implementation of control algorithms jointly governing motor actions and internal physiology.

The observation that many gut-responsive neurons also encode visual or motor signals suggests a more integrative role for these neurons in coordinating behavior than simply transmitting interoceptive cues. For instance, the zebrafish optic tectum, which here we report as containing cells responding to both visual and gut chemosensory stimulation, is the homologue of the mammalian superior colliculus (SC), a highly integrative region responding to a variety of sensory cues^85,86^. Though interoceptive signals have not yet been identified in OT cells in the zebrafish, many studies have shown that cells in the SC respond to interoceptive stimulation, with early results in cat SC estimating that over 40% of neurons in the SC are responsive to vagal stimulation^87^. This multimodal responsiveness implies that the brain does not process internal signals in isolation; instead, it integrates information about the body’s nutrient state with external sensory inputs to guide behavior adaptively. This finding aligns with the broader framework in behavioral neuroscience where internal states interact with external signals to modify complex behaviors^88,89^. By bridging the gap between interoception and exteroception, multi-sensory gut-responsive neurons could serve as critical nodes in the circuitry, ensuring that the animal’s behavior is finely tuned to both its internal needs and its external environment.

In conventional approaches, such as gut perfusion^4,8^, chemicals delivered into the gut lumen can be absorbed and enter the hepatic portal vein, making it difficult to distinguish the effects of luminal versus portal signals. Although our optical uncaging method enables precise spatial and temporal control of glucose release within the gut lumen, the released glucose can still be absorbed and reach the portal vein. To disentangle these overlapping effects, we directly introduced caged glucose into the bloodstream. Future work will identify the neural pathways that lead to the activation of the ventro-medial idMO during hepatic portal system glucose release but not during luminal glucose sensing, as well as determine the functional role of this region. Since not all blood glucose originates in the gut, this signal may enable the brain to estimate the physiological origin of glucose^70–72^. Together, this experimental design allowed us to separate the contribution of portal glucose from that of luminal glucose, representing a significant contribution beyond existing delivery methods.

Our paradigm also provides opportunities for studying the developmental time course of interoception. The fish’s small size and transparency permit whole-brain imaging in larval and juvenile zebrafish for the first several weeks of life. This presents the opportunity to investigate how gut innervation, gut-brain functional interactions, and the full repertoire of gut-related behaviors co-develop from hatching to maturity^90^. Applying our general paradigm to *Danionella cerebrum* would allow for the study of brain mechanisms of interoception in adults, as its small size and transparency persist into adulthood^91^. This allows for investigations of how interoceptive signals affect more complex cognitive behaviors that first appear in adult fish, including a variety of social behaviors^92^ and sophisticated learning and memory tasks^93^.

Overall, this platform enables investigations of interoceptive processing at high resolution. It opens opportunities for studying how internal bodily signals interact with sensory and motor circuits, how the brain maintains homeostasis, and how physiological states shape behavior. By offering a noninvasive, high-resolution, and versatile approach to manipulating and monitoring interoceptive signals, our system sets the stage for future research into the neural basis of brain–body communication.

## Methods

### Zebrafish

Larvae were reared at 28.5 °C under a 14:10-h light-dark cycle. Rotifers were provided as food during the rearing period. Zebrafish larvae aged 7–8 days post-fertilization (dpf) were selected for experiments. The transgenic line *Tg(elavl3:H2B-jGCaMP7f)*^jf90^ was used for all experiments. All experimental procedures were conducted in compliance with institutional animal care guidelines and were approved by the Institutional Care and Use Committee of Janelia Research Campus (JRC).

### UV–Vis spectroscopy

Spectroscopy was performed using 1-cm path length, 3.5-mL quartz cuvettes from Starna Cells or 1-cm path length, 1.0-mL quartz microcuvettes from Hellma. All measurements were taken at ambient temperature (22 ± 2 °C). Absorption spectra were recorded on a Cary Model 100 spectrometer (Agilent). Samples (50 μM) were prepared in phosphate-buffered saline (PBS), pH 7.4, for measurements.

### Analysis of photolysis products by tandem high-performance liquid chromatography–mass spectrometry (LC–MS)

To analyze photolysis products, solutions of caged-D-glucose or caged-L-glucose (200 μM) were prepared in PBS and placed in a glass vial. An aliquot of this freshly prepared solution was immediately analyzed by tandem high-performance liquid chromatography–mass spectrometry (LC–MS) using an Agilent 1200 LC–MS system equipped with an autosampler, diode array detector, and mass spectrometry detector (ESI; positive ion mode) using a 4.6 × 150 mm Gemini NX-C18 column with a 5–95% or 5–50% gradient of CH3CN in H2O containing constant 0.1% (v/v) TFA. Chromatograms were monitored using absorbance at 254 nm. The solution was then irradiated with 405 nm light from an LED array (LOCTITE CL20 flood array), followed by analysis using LC–MS.

### Synthesis and characterization of caged-D-glucose and caged-L-glucose

Caged-D-glucose was synthesized as previously described^41^. The enantiomer, caged-L-glucose, was synthesized using the same protocol but using L-glucose as the starting material. As expected, the enantiomeric pair of caged-D-glucose and caged-L-glucose show effectively identical 1H NMR spectra, 13C NMR spectra, absorption spectra, and photolysis traces (Supplementary Fig. 4). Caged-L-glucose: 1H NMR (CD_3_OD, 400 MHz) δ 8.07 (dd, *J* = 8.1, 1.3 Hz, 1H), 8.02 (dd, *J* = 7.9, 1.3 Hz, 1H), 7.71 (td, *J* = 7.6, 1.3 Hz, 1H), 7.56–7.46 (m, 1H), 5.28 and 5.08 (AB, JAB = 15.2 Hz, 1H), 4.43 (d, *J* = 7.6 Hz, 1H), 3.88 (dd, *J* = 11.9, 1.9 Hz, 1H), 3.68 (dd, *J* = 11.9, 5.2 Hz, 1H), 3.41–3.31 (m, 4H). 13C NMR (CD_3_OD, 101 MHz) δ 148.8 (C), 135.7 (C), 134.7 (CH), 130.3 (CH), 129.2 (CH), 125.5 (CH), 104.0 (CH), 78.1 (CH), 78.1 (CH), 75.2 (CH), 71.6 (CH), 68.4 (CH2), 62.7 (CH2). MS (ESI) calcd for C_13_H_19_NO_9_[M + H_2_O] + 333.1, found 333.0. To confirm these two compounds are enantiomers, the specific optical rotation of caged-L-glucose was measured to be [*α*]21D = 35 ± 1° (*c* = 0.1, pyridine). This is opposite in sign to the literature value of caged-D-glucose, which was measured to be [*α*]21D = − 36.4° (*c* = 0.55, pyridine).

### Uncaging light path

We built a custom UV light path in a light-sheet microscope setup to enable precise uncaging of compounds. The UV light source was a 7 W, 355 nm pulsed laser with a repetition rate of 2 kHz (3502-100, DPSS Laser Inc.). To control the UV intensity, a custom beam attenuator, consisting of a half-wave plate (WPHSM05-355, Thorlabs) mounted on a rotation mount (CRM05, Thorlabs), a polarized beam splitter (CCM1-PBS25-355-HP, Thorlabs), and a beam trap (BT600, Thorlabs), was placed within the laser path. The attenuated beam was directed onto a pair of galvo mirrors (1 and 2 in Supplementary Fig. 1, 6215K, Cambridge Technology), enabling independent horizontal and vertical positioning. The reflected beam was then directed through a scan-relay system (L1 in Supplementary Fig. 1, LA1509-ML, *f* = 100 mm and L2 in Supplementary Fig. 1, CA254-200-A-ML, *f* = 200 mm, Thorlabs) and focused onto the sample through the side light-sheet illumination objective (Obj 1 in Supplementary Fig. 1, 4×/0.28 NA, Olympus). The laser power at the sample plane was 0.7 mW. The UV beam had a point spread function (PSF) with a FWHM of ~4.5 μm (X/Y) and ~6 μm (Z) (Supplementary Fig. 2). To ensure precise UV beam positioning on the sample, a calibration pathway was implemented on the opposite side. This pathway included an opposite objective (Obj 4 in Supplementary Fig. 1, 4×/0.28 NA, Olympus), a lens (L4 in Supplementary Fig. 1, LA1509, Thorlabs), a shortpass filter (FESH0450, Thorlabs), and a camera (BFS-U3-32S4C-C, FLIR Integrated Imaging Solutions Inc.), enabling real-time visualization of the UV beam relative to the fish. Additionally, a NIR light source enabled visualization of the fish from the side using the same camera. This setup ensured accurate and reproducible UV beam delivery to the sample.

### Light-sheet microscopy

The light-sheet microscope and behavioral setup used in this study were designed as described previously^37^. Briefly, the detection pathway included a vertically mounted water-dipping objective (Obj 3 in Supplementary Fig. 1, 16×/0.8 NA, Nikon) attached to a piezo stage (P-725.4CD PIFOC, Physik Instrumente), along with a tube lens (TL3 in Supplementary Fig. 1, *f* = 200 mm) and an sCMOS camera (Orca Flash 4.0, Hamamatsu). Fluorescence separation was achieved using a band-pass filter (525/50 nm, Semrock) for GCaMP signals, effectively isolating them from scattered laser light (488 nm). Both illumination arms were equipped with galvanometer scanners (3–6 in Supplementary Fig. 1, 6210, Cambridge Technology), an f-theta lens (SL 1 and 2 in Supplementary Fig. 1, SL50-CLS2, *f* = 50 mm, Thorlabs), a tube lens (TL 1 and 2 in Supplementary Fig. 1, TTL200MP, *f* = 200 mm, Thorlabs), and an air illumination objective (Obj 1 and 2 in Supplementary Fig. 1, 4×/0.28 NA, Olympus). The galvanometer scanners and *f*-theta lens enabled lateral and axial scanning of the collimated laser beams (488 nm). To prevent direct retinal stimulation, the excitation laser was electronically switched off when it passed over the zebrafish eye.

### Estimation of imaging resolution

The spatial resolution of our imaging system was estimated using a parameter-free decorrelation analysis, as described by Descloux et al.^94^. Analyses were performed using the ImageDecorrelationAnalysis plugin for ImageJ. The parameters used for all analyses were: radius min = 0, radius max = 1, Nr = 50, and Ng = 10.

### Microgavage

The microgavage method used in this study was similar as in previous work by Cocchiaro and Rawls^43^. In brief, zebrafish larvae were anesthetized using tricaine, embedded in 3% methylcellulose (Sigma-Aldrich), and a fine borosilicate glass capillary (BF100-58-10, Sutter Instrument) cut to ~25–30 μm in diameter and smoothed using a microforge (Narishige MF2) was used to deliver approximately 4.6 nL of the desired substance into the gut lumen under a dissecting microscope. The concentration used for gavaging was 15 mM for caged glucose, 15 mM for caged glutamate, and 15 mM for caged L-glucose. Following the procedure, larvae were allowed to recover for 5–10 min until they resumed normal swimming behavior before the start of imaging experiments (Supplementary Fig. 3d–g). See Table 1 for source of key reagents.

**Table 1:** Reagents and resources

Reagent or resource	Source	Identifier
Chemicals, peptides, and recombinant proteins		
Caged D-glucose	This paper	
Caged L-glucose	This paper	
MNI-caged L-glutamate	Tocris	Cat# 1490
Tricaine	Sigma-Aldrich	Cat# A5040
Methylcellulose	Sigma-Aldrich	Cat# M 0387
Experimental models: organisms/strains		
Tg(elavl3:H2B-jGCaMP7f)	Yang et al.^39^	jf90
Software and algorithms		
FIJI	NIH Image	https://imagej.nih.gov/ij/
Adobe Illustrator	Adobe, San Jose, CA	https://www.adobe.com/products/illustrator.html
Custom code	This paper	https://github.com/benJames1212/gutBrainPipeline
Segmentation software	Mu et al.^62^	https://github.com/mikarubi/voluseg
ImageDecorrelationAnalysis	Descloux et al.^94^	https://github.com/Ades91/ImDecorr?tab=readme-ov-file

### Simultaneous optical uncaging in the gut and whole-brain light-sheet imaging

The preparation procedures followed previously established protocols^62^. Briefly, after recovering from microgavaging, non-paralyzed fish were embedded in 2% low-melting point agarose (Sigma-Aldrich) within a custom-made imaging chamber (CAD designs available upon request), and agarose was carefully removed around the head to allow for improved optical access during imaging, and around the tail for electrophysiological recordings. The position, onset and duration of UV were controlled by custom software developed in C# (Microsoft) during simultaneous whole-brain light-sheet imaging. Each UV pulse consisted of smaller, 1 ms long pulses separated by 5 ms of off time for a total of 200 ms. The UV power at the sample was ~0.7 mW. As the small beam diameter in our in vivo setup precluded direct HPLC-based quantification of photolysis efficiency, we instead quantified uncaging efficiency in vitro under controlled conditions. Specifically, we irradiated the same concentration of caged glucose with a 405 nm laser, which exhibits uncaging efficiency comparable to the 355 nm laser used in vivo (Supplementary Fig. 4b), at a power density of 0.897 mW/mm^2^ for 10 min. This resulted in ~80% photolysis, corresponding to an energy density of ~540 J/mm^2^. In comparison, our in vivo uncaging protocol (355 nm, 0.7 mW, ~16 µm^2^ spot size, 200 ms total exposure with a ~16% duty cycle) delivered an estimated energy density of ~1556 mJ/mm^2^, approximately 2.9-fold higher than the in vitro condition. Based on this comparison, we estimate that the degree of photolysis achieved in vivo exceeded 80%.

For behavioral experiments, spinal cord motor nerve signals were recorded. Extracellular recordings of the motor nerve signal were obtained using borosilicate glass pipettes (TW150-3, World Precision Instruments) fabricated with a vertical puller (PC-10, Narishige) and polished using a microforge (MF-900, Narishige) to achieve a tip diameter of ~40 µm. Recordings were conducted in current-clamp mode using an Axon Multiclamp 700B amplifier, with signals acquired via National Instruments data acquisition boards. Data were sampled at 6 kHz and band-pass filtered with a low-pass cutoff at 3 kHz and a high-pass cutoff at 100 Hz.

Visual stimuli were also simultaneously delivered through custom software developed in C# (Microsoft). Visual projections were made from below the fish using a miniature projector (Sony Pico Mobile Projector, MP-CL1) connected to the data acquisition computer, with the image projected onto a diffusive plastic screen affixed to the outside of the chamber, approximately 1 cm beneath the fish. The experiments were conducted in one-dimensional virtual environments comprising regularly spaced red and black bars (∼2 mm thick) to enable imaging of GCaMP-expressing fish. The stripes moved forward at a constant velocity, regardless of the fish’s swimming behavior, to ensure consistent visual stimulation throughout the experiment.

### Segmentation and time series extraction

Cells were segmented and time series extracted using a previously described volumetric segmentation software^62^. In brief, brain volumes were first registered to an average volume, and spatial footprints for cell segments were initialized by identifying local fluorescence maxima within temporally correlated and spatially continuous voxels. We then used a constrained non-negative matrix factorization to approximately solve the following equation using alternating least squares: $V\approx {WH}+{XI}$, where V is the (n x t) matrix of spatiotemporal fluorescence, W and H are the (n × c) and (c × t) matrices of spatial and temporal footprints of cell segments, and X and I are the (n x 1) and (1 × t) matrices of spatial and temporal traces of the background signal, and n, c, and t indicate voxels, cell segments, and time, respectively. Factorization of W and H was constrained and regularized to ensure spatial contiguity and sparsity, as well as mean fluorescence amplitude. ΔF/F was then computed as: $\varDelta F/F=\frac{{F}_{{raw}}-{F}_{{baseline}}}{{F}_{{baseline}}-{F}_{{background}}}$, with ${F}_{{baseline}}$ estimated from the 10th-lowest percentile of the raw fluorescence ${F}_{{raw}}$ over a rolling 5-min period, and ${F}_{{background}}$ computed from voxels located within the imaging volume but outside the brain. Note that as the UV laser induces a large artifact in frames recorded during stimulation, any frames recorded during UV stimulation are removed and replaced with a copy of the previous frame.

### Construction of brain maps

Functional volumes were registered to the Z-Brain atlas^95^ using the BigStream software package (https://github.com/JaneliaSciComp/bigstream). Following the registration of each fish onto a common reference brain, we transformed the centroids of each identified gut-responsive cell into a common anatomical space. We then expanded these centroids into cubes with side lengths of 5.5 µm in the x-y axes and 6 µm in the z-axis to minimize the effects of biological variability in brain anatomy and small errors in registration. In the regions containing the nodose ganglia, cubes measured 15 µm in the xy axes and 12 µm in the z-axis to accommodate larger registration error resulting from reduced imaging clarity at greater depths. The resulting volume was then smoothed with a Gaussian filter of standard deviation 1.624 µm.

### Regression analysis

In this work, regression fits were computed using smoothness-regularized distributed lag regression, where smoothness-regularized indicates a penalty on the magnitude of differences in temporally successive parameters^96^. As regressors, we used nutrient uncaging time series, visual stimulus time series, and behavioral motor activity time series. For a model taking a single time series, x, as input, a neuron’s response is modeled as ${y}_{t}={\sum }_{i=0}^{\rho }{\beta }_{i}{x}_{t-i}$, where y is a vector of neural activity of length T corresponding to the neuron’s activity across all T time points, ρ is the lag order that sets the length of time previous inputs are able to affect the current responses, and β is a vector of coefficients of length ρ. We did not use an offset term because △F/F is already offset-subtracted, and because it did not improve the prediction quality. To extend this regression to allow for the analysis of multiple cells simultaneously, vectors are converted to matrices. In matrix notation, Y=Xβ with (time × cells) matrix Y, (time × (ρ + 1)) matrix X, and coefficient matrix ρ of size ((ρ + 1) × cells). Note that the matrix X is constructed from a single time series, with each row shifted by one time point relative to the instantaneous time point.

We define a loss function:

1 $$L\left(X,Y;\beta \right)={{{\rm{\Vert }}}Y-X\beta {{\rm{\Vert }}}}_{2}^{2}+{\lambda }_{R}{{{\rm{\Vert }}}\beta {{\rm{\Vert }}}}_{2}^{2}+{\lambda }_{F}{{{\rm{\Vert }}}D\beta {{\rm{\Vert }}}}_{2}^{2}$$

incorporating an L2 penalty on both coefficient magnitudes (second term on the RHS in Eq. 1) and differences in temporally subsequent coefficient values to promote smoothness (third term on the RHS in Eq. 1, indexed by *t*). Here, D is a finite difference matrix (a matrix of size ((ρ + 1) × (ρ + 1)) with all diagonal entries equal to 2 and all one-off diagonal entries equal to negative one, penalizing the second derivative or curvature, see Supplementary Fig. 8a). Model parameters were selected to analytically minimize the loss function given in Eq. 1;

2 $$\hat{\beta }={\left({X}^{T}X+\psi \right)}^{-1}{X}^{T}Y$$

where $\psi=\,{\lambda }_{R}I+\,{\lambda }_{F}{D}^{T}D$, I is the identity matrix of size ((ρ + 1) × (ρ + 1)), ${\lambda }_{R}$ is the usual ridge regularization strength, and ${\lambda }_{F}$ is the regularization strength promoting temporal smoothness in kernels. Values for both regularization parameters were set manually (${\lambda }_{R}=2,\,{\lambda }_{F}=20$). For ρ>0, the resulting vector *β* can be viewed as a response kernel, while for ρ=0 the value of *β* is simply a coefficient.

Regressions taking multiple time series as input are performed analogously, with $X=[{X}_{1},\ldots,{X}_{N}]$ now being of size (time × ${\sum }_{i=1}^{N}({\rho }_{i}+1)$) constructed by concatenating each regressor ${X}_{i}$ with corresponding lag order ${\rho }_{i}$, and *β* now being a matrix of size (${\sum }_{i=1}^{N}({\rho }_{i}+1)$ × cells). For this work, lag orders were set to generate kernels approximately 55 s for control and gut UV pulse responses, 10 s for motor responses, and 5 s for visual responses, chosen by identifying the point at which each corresponding response decayed back to baseline. As not all imaging frequencies are a multiple of 60 s, the exact number of frames used in the lag response varies from fish to fish. Note that when multiple regressors are used in a single analysis, the finite difference matrix D is modified to prevent penalties occurring between the boundaries of distinct regressors (see Supplementary Fig. 8a), with each element ${D}_{{b}_{j},{b}_{j}+1}=0$ and ${D}_{{b}_{j}+1,{b}_{j}}=0$ for each boundary index ${b}_{j}\,={\sum }_{i=1}^{j}({\rho }_{i}+1)$, j=1,..,N-1. In other words, values of one-off diagonal elements of D occurring at the boundaries between regressors are set to zero, thereby “breaking” the temporal fusion of regressors and preventing a penalty between the last time point of one regressor and the first of another regressor. All regressions other than those used in Fig. 3 for estimating multi-modal activity were performed using leave-one-out cross-validation by leaving out periods of length 55 s beginning with each gut-targeted UV pulse. Regressions for estimating multi-modal activity were performed without cross-validation in order to utilize all data available. Regressors were zero-padded for ρ timesteps preceding neural activity in order to utilize all recorded experimental data.

### Gut-responsive cell selection

Gut-responsive cells were identified using the smoothness-regularized distributed lag regression model described above by creating a parts model. In this general paradigm, a “full” model is first constructed utilizing all available regressor data and then compared to a “part” model, which utilizes only a subset of the data incorporated in the “full” model. For cell selection, the “full” model consists of two regressors: the first corresponding to the time of gut-targeted UV pulses, and the second corresponding to the time of all UV pulses (thus incorporating off-gut UV pulses as well as on-gut UV pulses). The “part” model incorporates only a single regressor corresponding to the timing of all UV pulses, identical to the second regressor in the “full” model. Based on the results of the two models, cells are identified as responding to gut-nutrient uncaging if they meet the following criteria: the full model yields significant correlations (see below), has a statistically better fit than the “parts” model as assessed via a paired Wilcoxon signed-rank test between the difference in correlation between the “part” and “full” model for each gut-centered UV pulse, and the distribution of peak responses to gut pulses are statistically greater than the peak responses to control pulses, assessed via an unpaired Mann–Whitney test, with the last condition preventing false positives in the form of miniature UV evoked responses.

### Assigning threshold for significant correlations

We assume we can divide the set of recorded neurons into two mutually exclusive groups: those responsive to gut-nutrient uncaging and those unresponsive to gut-nutrient uncaging. As the activity of non-gut-responsive neurons by definition should not be predictable using gut-nutrient uncaging regressors, the distribution of correlations between these neurons and predictive models utilizing only gut-nutrient uncaging should be approximately normal with mean zero and variance set by noise. Gut-responsive neurons, on the other hand, should have a distribution of correlations centered at some value above zero, dependent upon the strength of a cell’s response to gut-nutrient uncaging. Consequently, analysis of the distribution of correlations across all cells in the brain yields a distribution heavy-tailed on the positive side corresponding to gut responses, while the negative side should be reflective of noise (Supplementary Fig. 8b). We thus constructed a null model by estimating the standard deviation, ${\sigma }_{G}$, of the left hand side of a Gaussian distribution centered on the mode, m, of the data. We then utilized the parameters of this null model to set the threshold for significant correlations as 3 × ${\sigma }_{G}+m$. Assuming the parameters of the null model were correctly estimated, this results in a set false-positive rate of 0.13%, equivalent to a total of 195 cells falsely identified as gut-responsive out of a total of 150,000 cell segments identified in an experiment.

### Signal and noise correlation analysis

In order to compute noise and signal correlations between neurons in response to gut-nutrient uncaging, we first extracted trial responses ${x}_{{itk}}$ for each gut-responsive neuron i, time point t, and trial number k. Signal correlations, $({r}_{{S}_{i,j}})$, were then computed for each pair of neurons computing the Pearson correlation between trial averaged responses: ${r}_{{S}_{i,j}}={corr}_{t}({ < x}_{{itk}}{ > }_{k}, < {x}_{{jtk}}{ > }_{k})$ for neurons i and j, where $< \cdot \,{ > }_{k}$ is the average over trials. Noise correlations between pairs were computed by averaging the correlation of each neuron’s trial residuals (trial response – averaged response) over all T trials: ${r}_{{N}_{i,j}}=\frac{1}{K}{\sum }_{k=1}^{K}\left[{corr}_{t}\left({x}_{{itk}}-{ < x}_{{itk}}{ > }_{k},{x}_{{jtk}}- < {x}_{{jtk}}{ > }_{k}\right)\right]$. Matrices (as in Fig. 4b, c) were then constructed from these analyses by averaging all data from distinct neuron pairs within and between each brain region.

### Quantification and statistical analysis

Error bars displayed in the figures represent the standard error of the mean (SEM). Statistical analyses were performed using the SciPy Stats package.

### Reporting summary

Further information on research design is available in the Nature Portfolio Reporting Summary linked to this article.

## Supplementary information

Supplementary Information

Peer Review File

Reporting Summary

Description of Additional Supplementary Files

Supplementary Video 1

## Data Availability

All datasets are deposited to Figshare: https://doi.org/10.25378/janelia.31967640.

## Code availability

All original code has been deposited at GitHub and is publicly available: https://github.com/benJames1212/gutBrainPipeline. Any additional information required to reanalyze the data reported in this paper is available from the lead contact upon request.
