# Author Correction: Whole-brain, all-optical interrogation of neuronal dynamics underlying gut and vascular interoception in zebrafish

**DOI:** 10.1038/s41467-026-78324-z

**Published:** 2026-10-01

**Authors:** Weiyu Chen, Ben James, Virginie M. S. Ruetten, Sambashiva Banala, Ziqiang Wei, Xueying Yang, Greg Fleishman, Igor Siwanowicz, Mikail Rubinov, Jeremy Delahanty, Mark C. Fishman, Florian Engert, Maneesh Sahani, Luke D. Lavis, James E. Fitzgerald, Misha B. Ahrens

**Affiliations:** 1 Janelia Research Campus, Howard Hughes Medical Institute, Ashburn, VA USA; 2 Department of Neurobiology, Northwestern University, Evanston, IL USA; 3 Gatsby Computational Neuroscience Unit, University College London, London, UK; 4 Department of Biomedical Engineering, Vanderbilt, Nashville, TN USA; 5 Harvard Stem Cell Institute, Harvard University, Cambridge, MA USA; 6 Department of Molecular and Cellular Biology, Harvard University, Cambridge, MA USA; 7 NSF-Simons National Institute for Theory and Mathematics in Biology, Chicago, IL USA

**Keywords:** Neuroscience, Physiology, Neural circuits, Peripheral nervous system

Correction to: *Nature Communications* https://doi.org/10.1038/s41467-026-76242-8, published online 27 August 2026

In the version of the article initially published, in the “Signal and noise correlation analysis” section of the Methods, “$< \,\cdot \,{ > }_{t}$” should have read “$< \,\cdot \,{ > }_{k}$”. This correction has been made to the HTML and PDF versions of the article.

