# Supplementary material for: Whole-brain, all-optical interrogation of neuronal dynamics underlying gut and vascular interoception in zebrafish
Source: Nat Commun. 2026 Aug 27;17:9881. doi: 10.1038/s41467-026-76242-8 (PMC13583002; doi:10.1038/s41467-026-76242-8)
Supplement: Supplementary file 1 [file 41467_2026_76242_MOESM1_ESM.pdf]

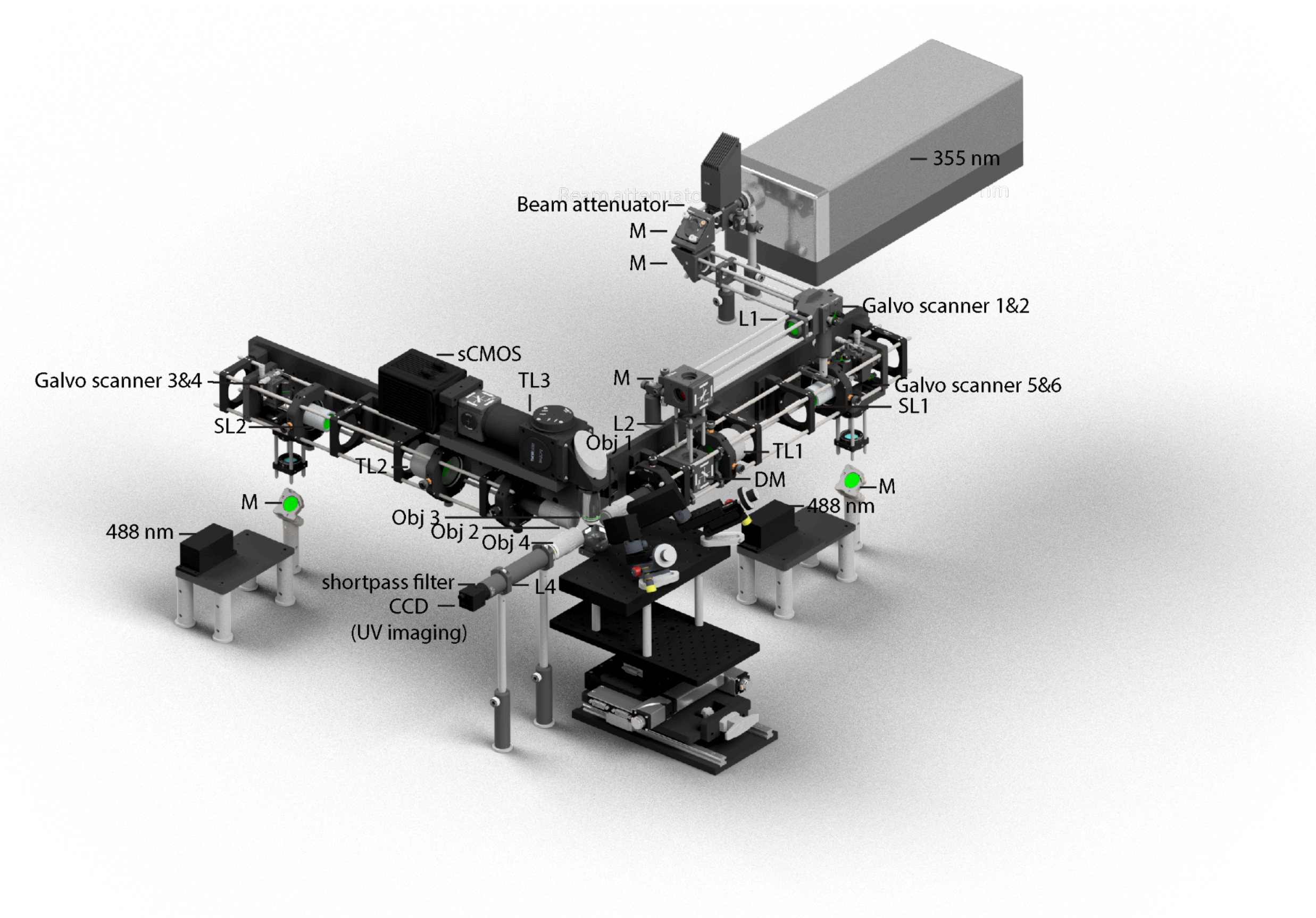

Supplementary Fig. 1. Microscope setup.
Schematic of the integrated UV uncaging path and light-sheet imaging system. A 3 W, 355 nm DPSS laser is attenuated by a custom beam attenuator at the laser head, then directed onto a pair of galvanometer scanners (1 & 2) for independent horizontal and vertical scanning. After passing through a telescope (L1 & L2), the UV beam is focused onto the sample via the light-sheet objective. A calibration pathway on the opposite side uses a lens (L4) and calibration camera to monitor the beam in real time, ensuring precise alignment and delivery of UV light to the gut. The light-sheet microscope is designed as described previously[17] and features a vertically mounted water-dipping detection objective (obj3, 16×/0.8 NA, Nikon) on a piezo stage, which projects fluorescence onto an sCMOS camera (Orca Flash 4.0, Hamamatsu). GCaMP emission is isolated by a 525/50 nm band-pass filter (525/50 nm, Semrock). Two horizontal illumination arms, each with an air objective (Obj 1 & 2, 4×/0.28 NA, Olympus), tube lens (TL 1 & 2), f-theta lens (SL 1 & 2), and galvanometer scanners (3-6, Cambridge Technology), generate the 488 nm excitation light-sheet, which is electronically shuttered to avoid stimulating the zebrafish eye. M, mirror; SL, scan lens; TL tube lens; DM, dichroic mirror; L, lens; Obj, objective.

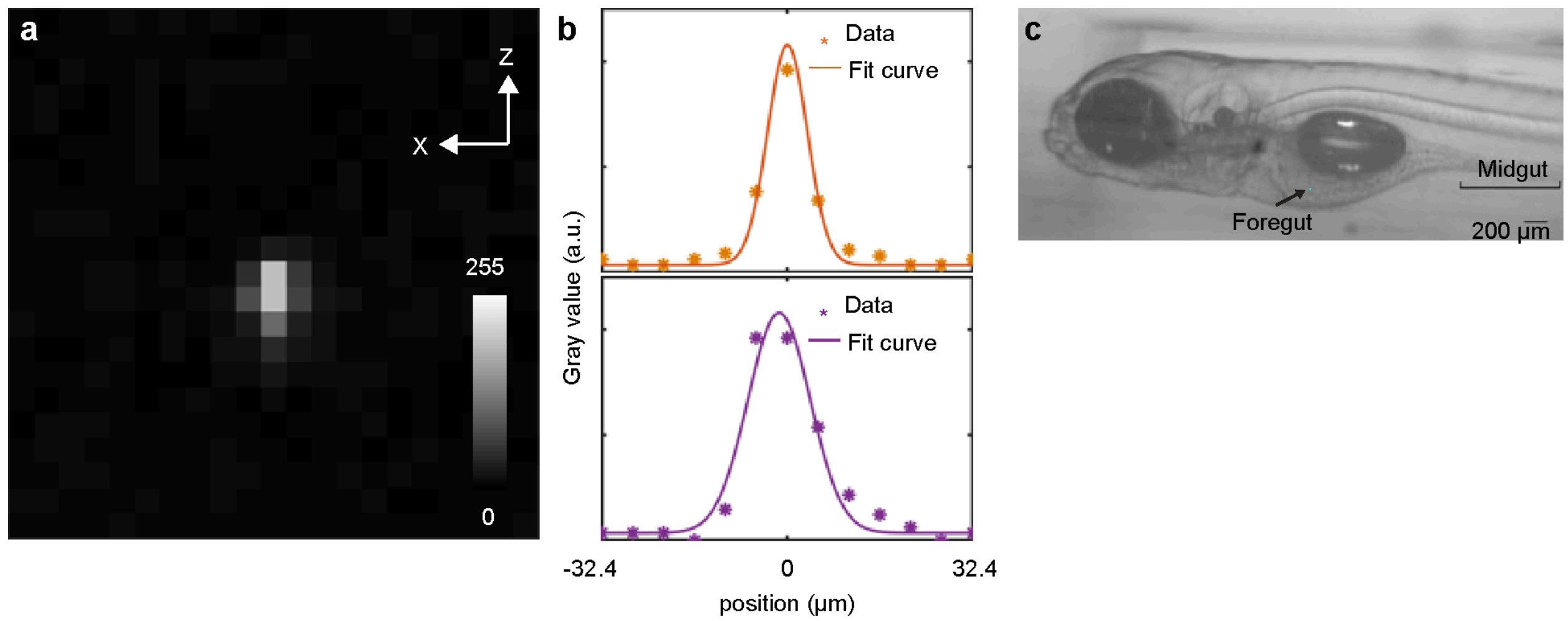

Supplementary Fig. 2. Characterization of UV Beam Profile and Imaging Resolution in the Light-Sheet System.

a The beam profile at the focal plane of the illumination plane was measured with a CMOS camera (IDS UI-1240LE-M-GL, E2v).

b 1D PSF plots in X direction (up) and Z direction (bottom). Beam profile was fitted with a 2D gaussian function. The full-width-at-half-maximum of the PSFs in X and Z directions were calculated as 3.5 μm and 5.4 μm, respectively.

c Lateral view of a 7 dpf larval zebrafish showing the positions of the foregut and midgut for comparison with the size of the UV beam profile. The tiny green dot indicated by the arrowhead is not the actual UV beam, but an artificially added marker used solely to illustrate the relative scale of the UV beam in comparison to the dimensions of the foregut and midgut.

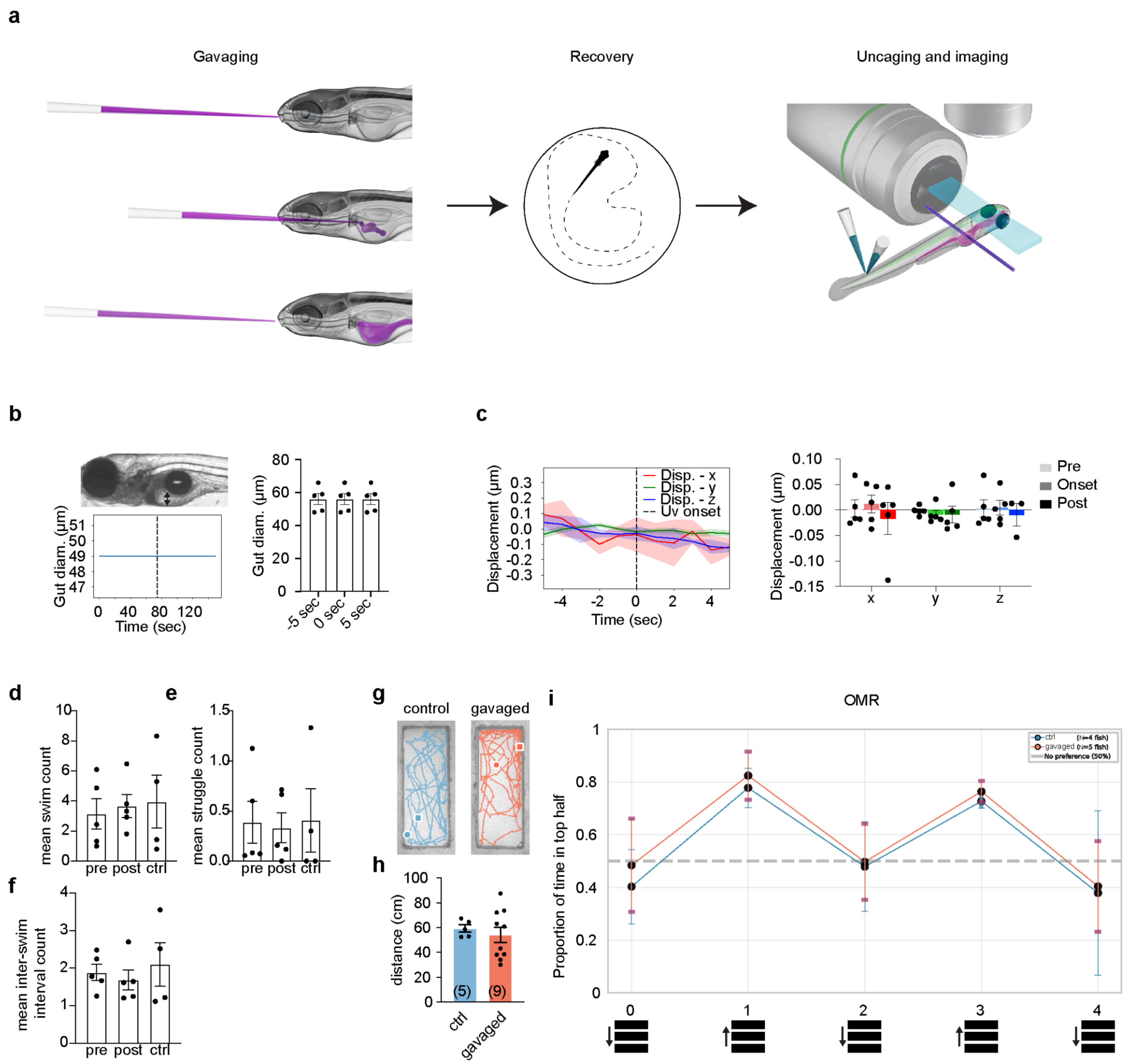

Supplementary Fig. 3. Experimental overview.

a Overview of the experimental procedure. After gavaging caged compounds into the larval zebrafish gut (left), the fish recover in a Petri dish (middle) for 5–10 minutes until they resume normal swimming behavior, before being transferred to the light-sheet microscope for uncaging and imaging (right).

b Chemical stimulation of the gut without mechanical dilation. Time-lapse measurements of gut diameter (left) show no discernible changes during uncaging, indicating minimal mechanical influence. Summary data (right) showing the gut diameter remains consistent before and after stimulation (mean ± SEM; N = 5 fish).

c Optical uncaging maintains tissue stability during imaging. Displacement in the x, y, and z axes (left) remains near baseline across pre-, onset-, and post-uncaging intervals, as shown by the summarized bar chart (right) (mean ± SEM; N = 5 fish).

d Summary data showing the mean swim count from fictive swimming recordings (mean ± SEM). pre: baseline period before uncaging; post: 5 seconds after uncaging; ctrl: control trials in which UV light was targeted outside the fish. N = 5 fish, pre and post; N = 4, control.
e Summary data showing the mean struggle count from fictive swimming recordings (mean ± SEM). N = 5 fish, pre and post; N = 4, control.
f Summary data showing the mean inter-swim interval count from fictive swimming recordings (mean ± SEM). N = 5 fish, pre and post; N = 4 fish, control.
g Representative traces of spontaneous swimming in control and gavaged fish. The square marks the starting position and the dot marks the ending position.
h Quantification of total distance traveled during spontaneous swimming (mean ± SEM; N = 5 fish for control; N = 9 fish for gavaged group).
i Optomotor response (OMR) behavior in control and gavaged fish. Moving gratings were presented once every minute, each lasting 1 minute, with motion alternating between upward and downward directions in the chamber. The proportion of time spent in the top quadrant was calculated by dividing the chamber into equal top and bottom regions and measuring the percentage of time spent in the top region within each minute (mean ± SEM; N = 4 fish for control; N = 5 fish for gavaged group).

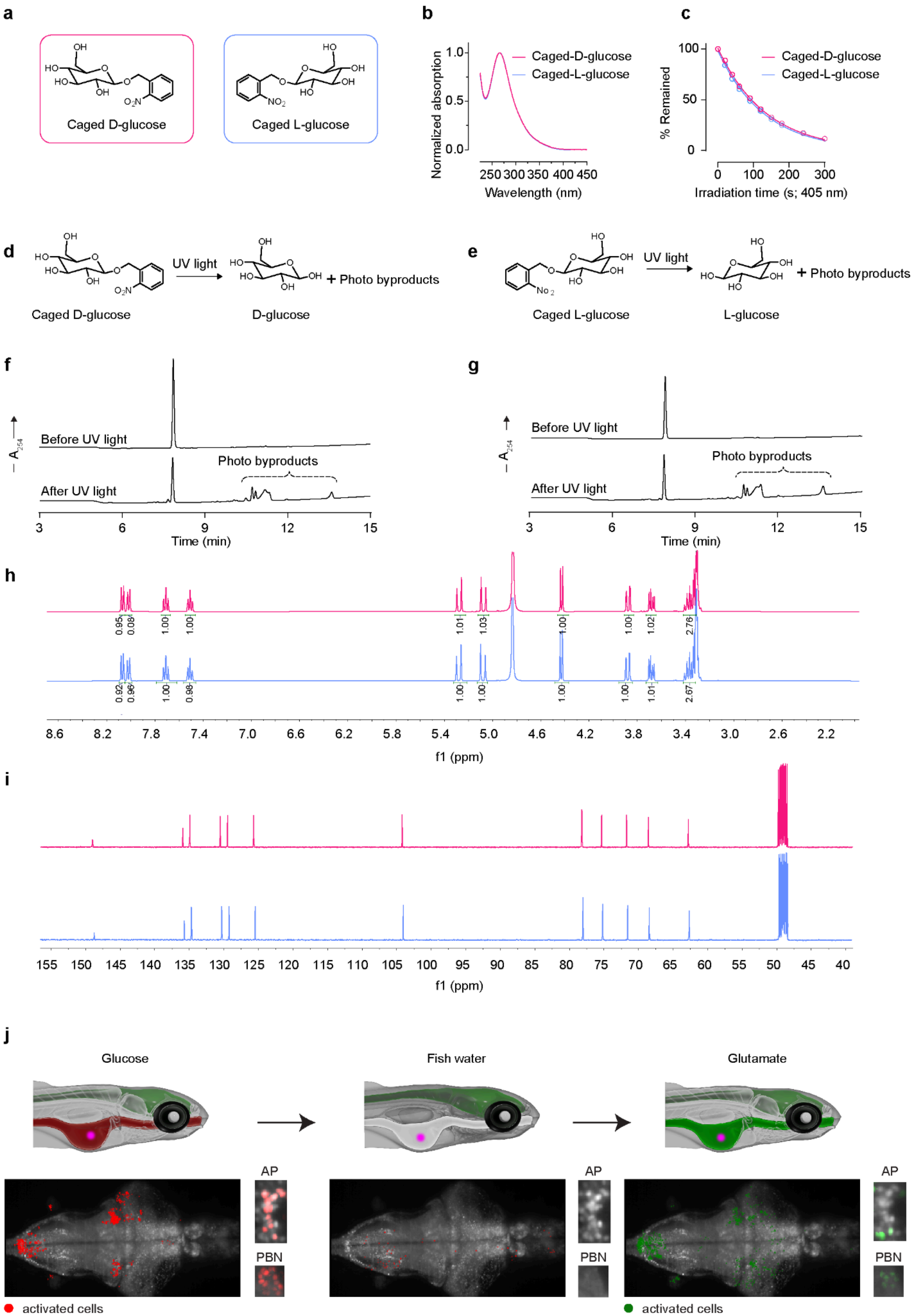

a
Caged D-glucose
Caged L-glucose
b
Normalized absorption
Caged-D-glucose
Caged-L-glucose
Wavelength (nm)
c
% Remained
Caged-D-glucose
Caged-L-glucose
Irradiation time (s; 405 nm)
d
UV light
+ Photo byproducts
Caged D-glucose
D-glucose
e
UV light
+ Photo byproducts
Caged L-glucose
L-glucose
f
Before UV light
After UV light
Photo byproducts
Time (min)
g
Before UV light
After UV light
Photo byproducts
Time (min)
h
f1 (ppm)
i
f1 (ppm)
j
Glucose
Fish water
Glutamate
AP
PBN
activated cells

Supplementary Fig. 4. Chemical characterization of caged D- and L-glucose and control uncaging strategy.

a Chemical structure of caged D-glucose and caged-L-glucose.

b Normalized absorption spectra for caged D- and L-glucose. Note that caged D- and L-glucose have effectively identical absorption spectra.

c Photolysis conversion vs. irradiation time for caged D- and L-glucose. Note that caged D- and L-glucose have effectively identical photolysis traces.

d Chemical uncaging operation for caged D-glucose

e Chemical uncaging operation for caged L-glucose.

f,g HPLC chromatograms of caged D- and L-glucose before and after irradiation. For experiments B-E, samples were prepared in PBS and irradiated with 405 nm LED (LOCTITE CL20 flood array). Chromatograms show caged-D-glucose and caged-L-glucose release of same photo byproducts.

h ^{1}H NMR of caged-D-glucose (magenta) and caged-L-glucose (blue) recorded in CD_3OD.

i ^{13}C NMR of caged-D-glucose (magenta) and caged-L-glucose (blue) recorded in CD_3OD. Note that caged-D-glucose and caged-L-glucose exhibited effectively identical ^{1}H NMR spectra, ^{13}C NMR spectra, indicating that the two chemicals share the same electronic environment. However, chirality measurement revealed a difference: caged-L-glucose had an optical rotation of $[\alpha]^{21}_{D}$ = 35±1° (c =0.1, pyridine), which is opposite of the literature value of caged-D-glucose, $[\alpha]^{21}_{D}$= −36.4° (c =0.55, pyridine). This opposite optical rotation confirms that the two chemicals are indeed an enantiomeric pair.

j Example case showing how multiple compounds can be introduced consecutively into the same fish to compare nutrients‐evoked responses. In this example, glucose (left), fish water (middle), and glutamate (right) are sequentially delivered to the gut by microgavaging multiple times in the same fish.

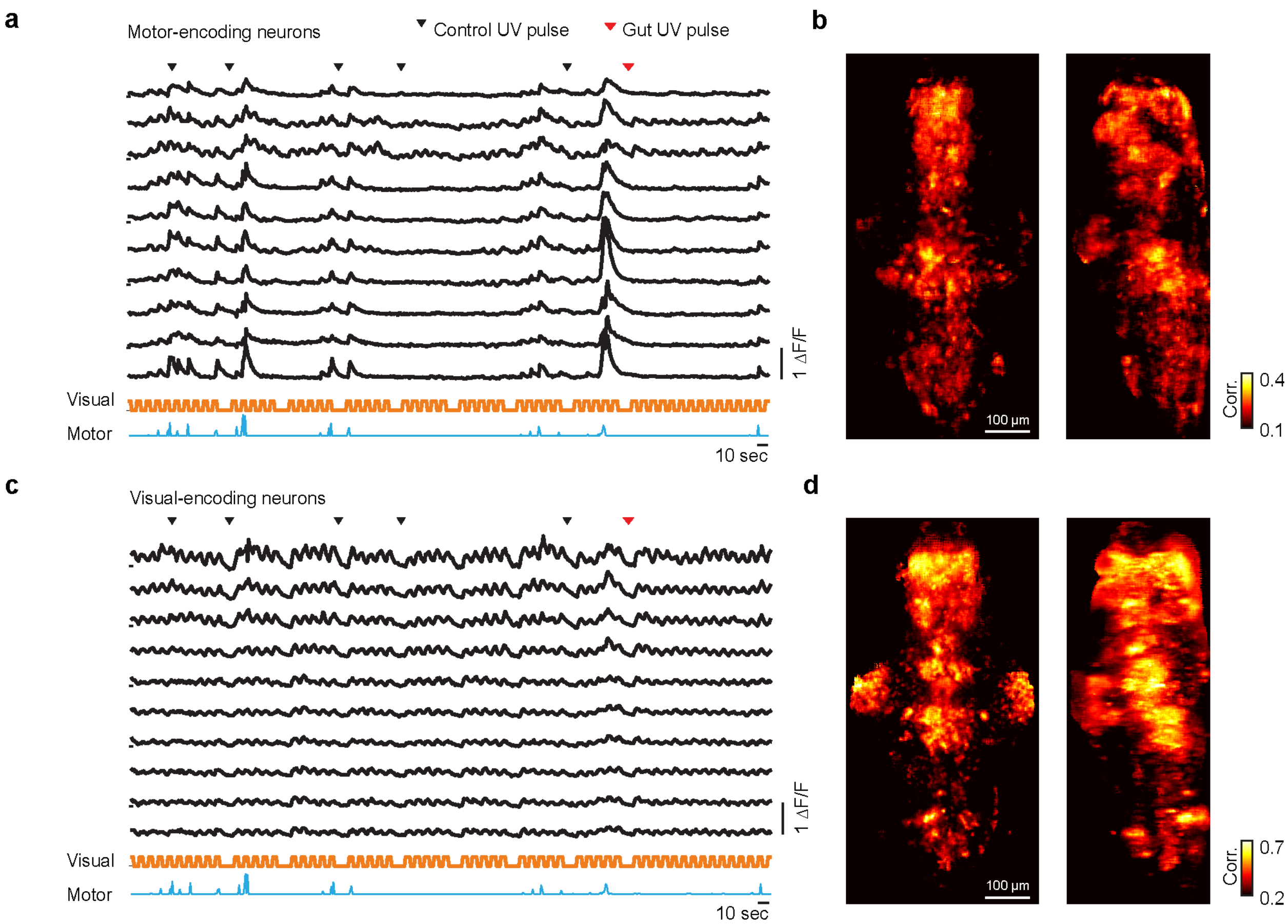

Supplementary Fig. 5. Brain-wide detection of visual- and motor-responsive cells.
a Example calcium traces of motor-responsive neurons. Each row represents the activity of a single neuron. The orange trace indicates visual motion, with upward segments representing forward movement of the visual gratings and downward segments indicating no motion. The blue trace reflects motor power derived from a sliding window of the tail voltage signal. The black triangles mark the onsets of control UV stimulation (UV targeted outside of the fish), and the red triangle indicates onset of gut stimulation.
b Average brain map showing the spatial location of motor-related neurons. Left: dorsal view. Right: sagittal view. Colors represent the correlation coefficient between neuronal activity and motor signals (N = 6 fish).
c Example calcium traces of visual-related neurons.
d Average brain map showing the spatial location of visual-responsive neurons (N = 6 fish).

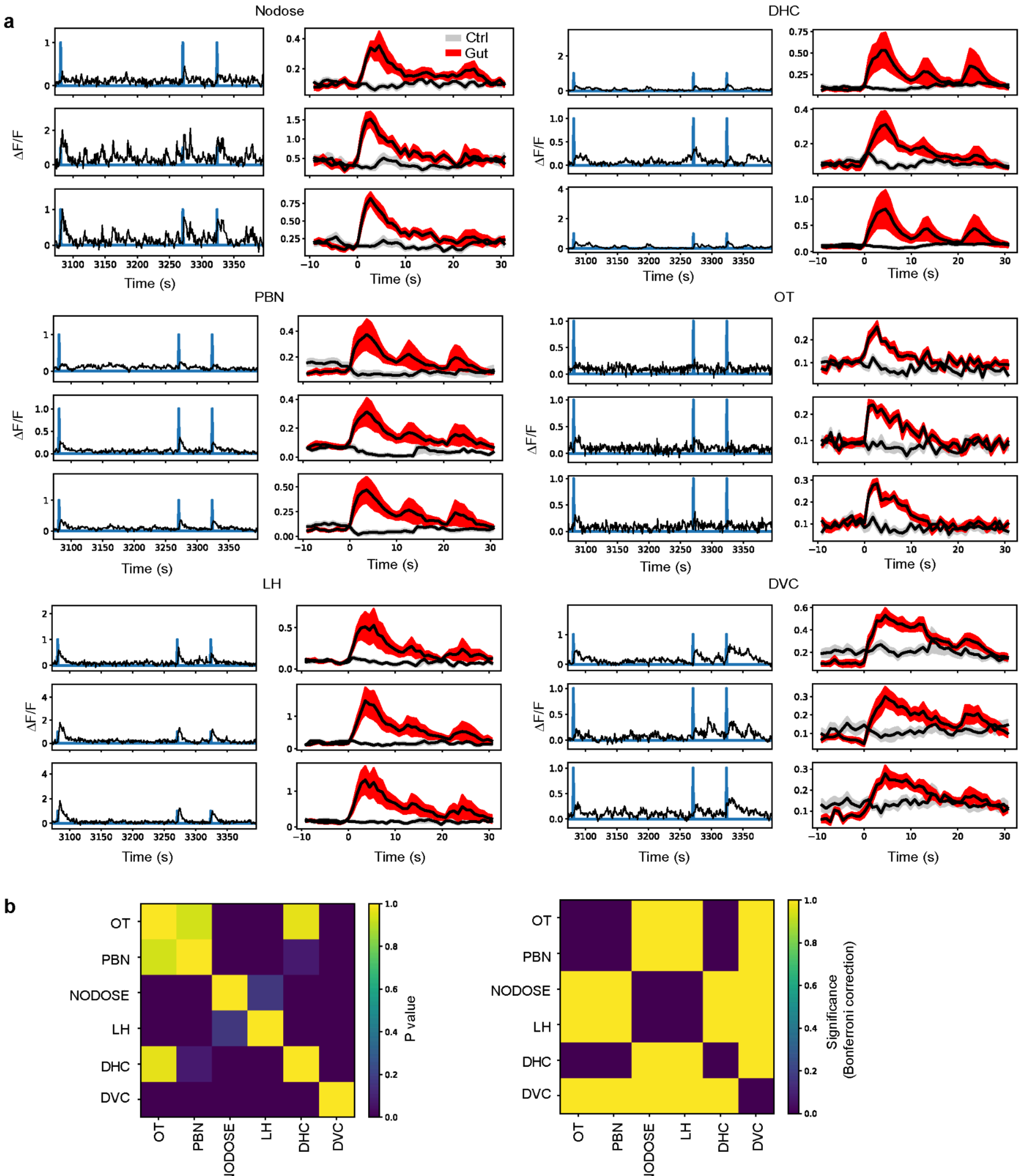

Supplementary Fig. 6. Noise Correlation Traces and Timescale Significances.
a Example time series in response to gut stimulation (blue lines) (left) and averages (right) to gut and control UV pulses for a selection of cells in various brain regions. Note that nodose, more than any other brain region, contains neurons with gut responses consisting of multiple events. Pooling from multiple units may smooth these responses

in the brain, reducing noise correlations between nodose cells and cells within the brain, as seen in Fig. 4c.

b Matrix of p-values for rank sum tests of differences in FWHM, as seen in Fig. 4f (left), and results of Mann-Whitney significance tests after Bonferroni correction (right). Values of elements equal to one indicate statistical significance.

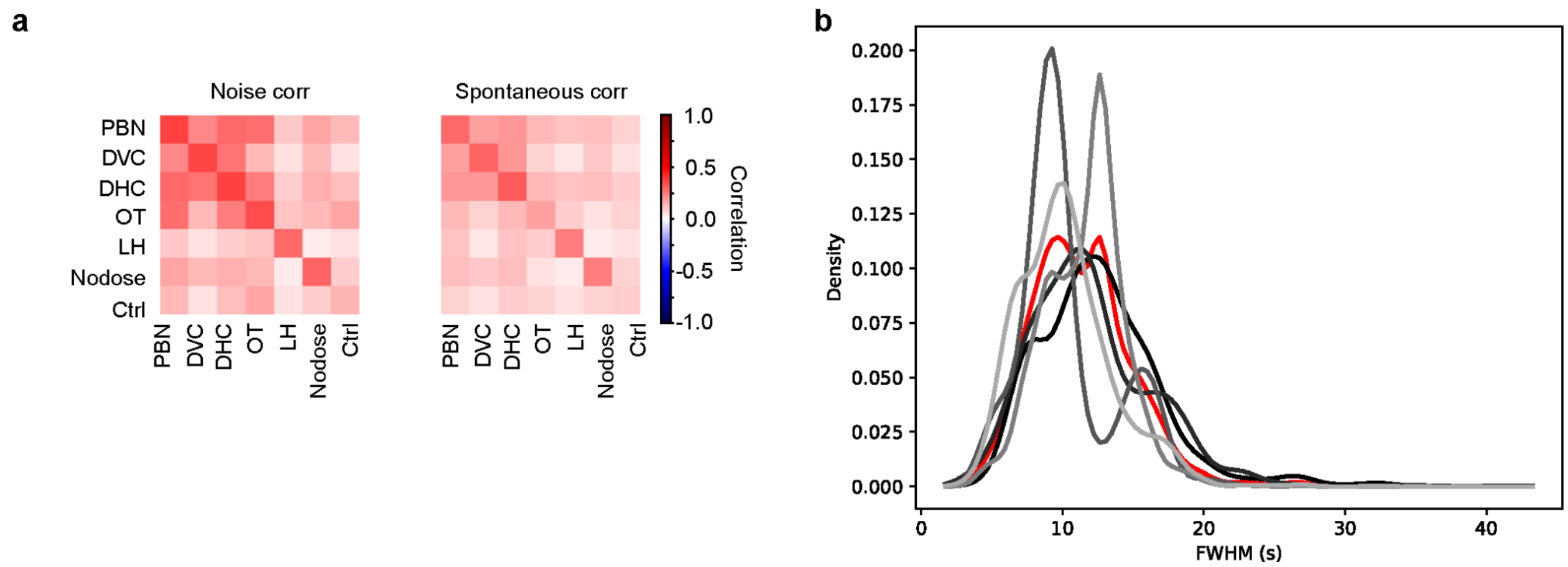

Supplementary Fig. 7. Total correlation Spatial Maps and Distributions of FWHM Across Individual Fish.

a Mean noise correlation between identified gut-sensitive cells by brain region, as in **Fig. 4b** (left). Same as left, but for total correlation during spontaneous, non-gut stimulated regions (right).

b Distributions of FWHM for individual fish (grayscale) and the across-fish average distribution (red) for selected brain regions.

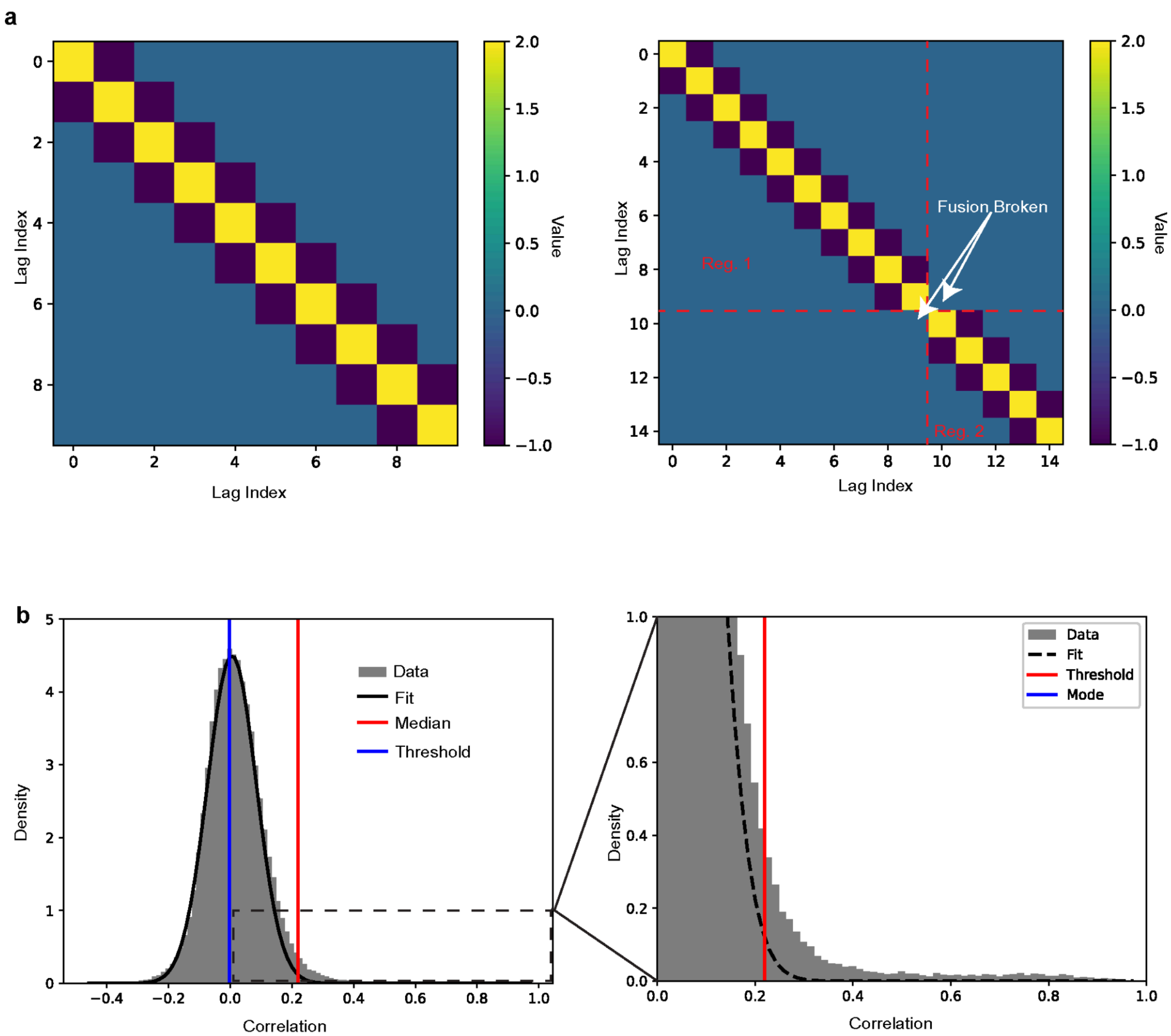

Supplementary Fig 8. Methods demonstration.

a Left: a finite difference matrix for a single regressor of lag order 10. Right: a modified finite difference matrix for two regressors (shown as boxes in red dashed lines). Regressor 1 has a lag order of 10, and regressor 2 has a lag order of 5. Fusion between the two regressors is broken by setting $D_{b_j,b_j+1} = 0$ and $D_{b_j+1,b_j} = 0$ for all boundary indices b_j.

b Left: the distribution of correlations between each neuron and its best fit linear model (gray) was used to construct a null model for assessing whether a neuron has a statistically significant response to gut stimulation. Data from the left of the mode (blue line) was used to fit the left side of a Gaussian distribution (black line). The threshold for significant responses was then set to the mode plus three times the estimated standard deviation (red line). Right: blow-up of the box in left highlighting the presence of neuron correlations above the computed threshold.

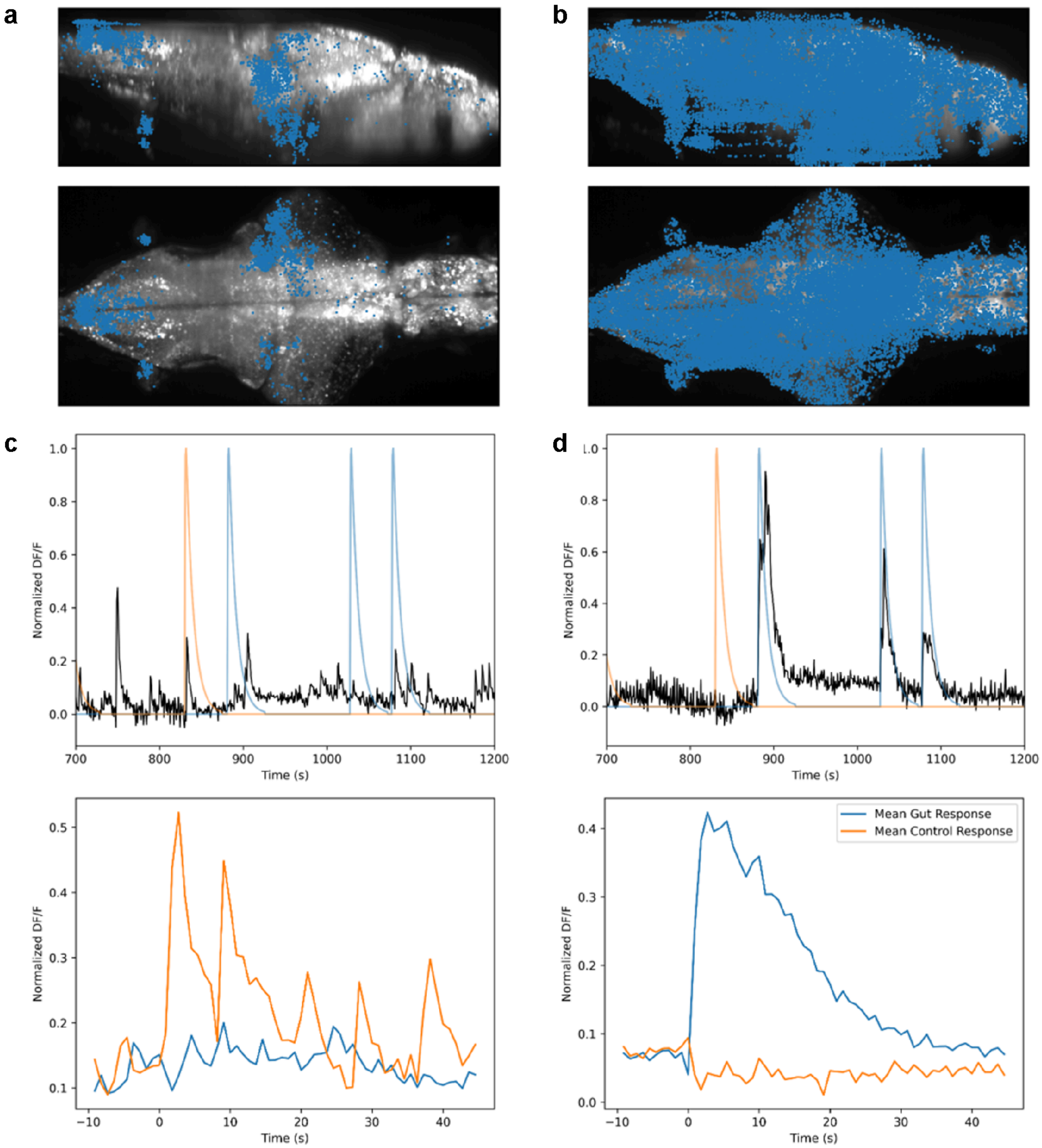

Supplementary Fig 9. Comparison to vanilla method for gut-sensitive neuron identification as described in Methods - Regression Analysis.

a Brain maps produced from selected cells for a single fish for the FLR method.

b Same as (a), but for the vanilla method (see Methods - Regression Analysis). Note the lack of clear spatial segregation for the vanilla, but not the FLR method.

c Top: ΔF/F time series for a cell falsely selected as gut sensitive with the vanilla method. Bottom: average responses to control and gut stimulation for the same cell.

Note the limited synchrony between gut stimulation and response. Cell was identified as significantly correlated to the experimental regressor ($p < 0.001$) and not significantly correlated to the control regressor ($p = 0.28$).

d Same as (b), but for a cell falsely selected as non-gut sensitive. Cell was identified as significantly correlated to the experimental regressor ($p < 0.001$), but also significantly correlated to the control regressor ($p = < 0.001$).
