# Supplementary material for: Whole-brain, all-optical interrogation of neuronal dynamics underlying gut and vascular interoception in zebrafish
Source: Nat Commun. 2026 Aug 27;17:9881. doi: 10.1038/s41467-026-76242-8 (PMC13583002; doi:10.1038/s41467-026-76242-8)
Supplement: Supplementary file 2 [file 41467_2026_76242_MOESM2_ESM.pdf]

nature portfolio

Peer Review File

Whole-brain, all-optical interrogation of neuronal dynamics underlying gut and vascular interoception in zebrafish

Corresponding Author: Dr Misha Ahrens

This file contains all reviewer reports in order by version, followed by all author rebuttals in order by version.

Version 0:

Reviewer comments:

Reviewer #2

(Remarks to the Author)
In this revised manuscript, the authors have addressed my critiques in a satisfying way - either providing new data and analyses, or providing written justification about experimental design choices or confounds. The expansion to uncaging in the circulatory system is a major new addition, and expands the usefulness of this technique. I have no further comments, and am supportive of publication.

(Remarks on code availability)

Reviewer #3

(Remarks to the Author)
The revised manuscript by Chen et al. has been substantially improved, and the authors have addressed my initial concerns point by point. The study now presents a well-demonstrated framework and methodology for all-optical interrogation of whole-brain activity during interoception, and I support its publication.

Lamiae Abdeladim

(Remarks on code availability)

Reviewer #4

(Remarks to the Author)
I thank the authors for addressing the comments and improving the manuscript. While interesting, the new results seem to indicate a generic response to elevated blood glucose, and less a gut specific response. Could the difference in response profile between gut and portal vein be due to the flow of circulation vs gut peristalsis, leading to a different amount of irradiation or caged glucose exposed to the pulse?

The authors addressed some of my comments. However, for the response regarding Fig.1E, I assume they count an area as 6/6 if only a single voxel is excited 6 times? Some form of ratio of activation may be more relevant as the DVC is a lot more activated than the OT for example, and it seems that those metrics do not represent the data in a useful manner.

Regarding the passive state, what do the authors mean by many respond consistently? Could we have some quantitative assessment? And/or a comparison between high motor and low motor states, which I assume would be the best proxy for active vs passive state?

With their response to reviewer 2 point 5 (or 3 point 4) about the lagged regression, could we have the threshold used in the figure of their response? As well as the correlation coefficients from C and D. It seems strange that the “vanilla” method would have such a large difference in the cells identified by their kernel, and a more quantitative comparison would allow us to better understand the differences and if it is a thresholding problem.

(Remarks on code availability)

Open Access This Peer Review File is licensed under a Creative Commons Attribution 4.0 International License, which permits use, sharing, adaptation, distribution and reproduction in any medium or format, as long as you give appropriate credit to the original author(s) and the source, provide a link to the Creative Commons license, and indicate if changes were made.
In cases where reviewers are anonymous, credit should be given to 'Anonymous Referee' and the source.
The images or other third party material in this Peer Review File are included in the article's Creative Commons license, unless indicated otherwise in a credit line to the material. If material is not included in the article's Creative Commons license and your intended use is not permitted by statutory regulation or exceeds the permitted use, you will need to obtain permission directly from the copyright holder.
To view a copy of this license, visit https://creativecommons.org/licenses/by/4.0/

Response to Reviewers

We thank the Reviewers and Editors for their thoughtful feedback and for carefully considering our manuscript. Below, we provide replies to the final comments of Reviewer 4.

Reviewer #4 (Remarks to the Author):

I thank the authors for addressing the comments and improving the manuscript. While interesting, the new results seem to indicate a generic response to elevated blood glucose, and less a gut specific response. Could the difference in response profile between gut and portal vein be due to the flow of circulation vs gut peristalsis, leading to a different amount of irradiation or caged glucose exposed to the pulse?

We thank the Reviewer for this thoughtful comment. We varied the irradiation duration during gut uncaging to change the amount of glucose released. While increasing irradiation increased the amount of uncaged glucose, it did not alter the set of brain regions that were activated. In particular, we did not observe differential activation of the idMO under these conditions. Instead, the primary effect of increasing the dose was a change in the number of neurons activated within already responsive regions, rather than the recruitment of new regions. This dose-dependent increase in the number of activated neurons is shown in Fig. 2H-I. These results suggest that differences in irradiation exposure or glucose dose are unlikely to explain the distinct activation patterns observed between gut and portal vein stimulation.

The authors addressed some of my comments. However, for the response regarding Fig.1E, I assume they count an area as 6/6 if only a single voxel is excited 6 times? Some form of ratio of activation may be more relevant as the DVC is a lot more activated than the OT for example, and it seems that those metrics do not represent the data in a useful manner.

We thank the Reviewer for this helpful comment and for the opportunity to clarify the metric used in Figure 1E. The “6/6” value indicates that a given brain region contained responsive cells in all six fish, rather than reflecting repeated activation of a single voxel.

Our imaging voxel size is 0.81 × 0.81 × 2 µm, whereas the highlighted regions in Figure 1E (dashed circles) span approximately 57 µm in diameter. As shown in the brain maps in Figure 1E, the activated regions consist of clusters of responsive cells that fill these

areas. Therefore, regions labeled as 6/6 represent consistent activation across animals at the regional level, not activation of a single voxel across trials.

Regarding the Reviewer's suggestion to amplitude metrics, we note that the amplitudes of gut-evoked responses are comparable between OT and DVC (Fig. 1G). The variability in the spatial maps outside of PBN and DVC reflects either subtle differences in activation patterns between animals or increased registration error at greater imaging depths, as described in our manuscript.

We chose to visualize the spatial maps of responsive cells rather than raw amplitude values because spatial maps ensure that observations are robust across multiple animals, effectively filtering out noise and single-subject false positives. Amplitude measurements can be confounded by varying cell properties, such as calcium buffering, across different cell types. This approach of identifying brain regions based on the spatial distribution of responsive cells across animals follows established practices in whole-brain zebrafish imaging studies (e.g., Marques et al., 2020).

Regarding the passive state, what do the authors mean by many respond consistently? Could we have some quantitative assessment? And/or a comparison between high motor and low motor states, which I assume would be the best proxy for active vs passive state?

In our original response, we stated "As with Mu et al., 2019, under open-loop conditions, fish may transition between active and passive states during the experiment. Trial-by-trial analysis shows that many gut-responsive cells respond consistently across trials, regardless of possible behavioral state transitions". When stating this we did not intend to make any strong claims about differences in cell activity between potential passive and active states, but rather that responses to gut stimulation seem qualitatively consistent across trials in gut-sensitive cells. While it would indeed be interesting to have a more quantitative assessment of passive vs. active state on the responsivity of gut-sensitive neurons, we are particularly hindered by the amount of gut stimuli we can present to a single fish. The experiments performed by Mu et al., 2019 consisted of hours-long recordings with thousands of trials of closed or open loop visual stimulation, allowing for identification of differences with a small effect size. Our experiments, on the other hand, utilize much shorter experiments consisting of a maximum of approximately 10 gut stimuli and only dozens of trials of closed or open loop stimulation. Consequently, any thorough quantification comparing responses to gut stimuli in passive vs. active state would have minimal statistical power.

With their response to reviewer 2 point 5 (or 3 point 4) about the lagged regression, could we have the threshold used in the figure of their response? As well as the correlation coefficients from C and D. It seems strange that the “vanilla” method would have such a large difference in the cells identified by their kernel, and a more quantitative comparison would allow us to better understand the differences and if it is a thresholding problem.

We apologize for being unclear. The cell selection process for the vanilla method presented in the previous response utilized t-tests on the correlation between a cell’s recorded activity and the convolved stimuli rather than a threshold on the magnitude of the correlation. We thus took as responsive cells those with a significant correlation to the experimental convolved stimuli trace ($p_{Exp} > \alpha$) and a non-significant correlation to the control convolved stimuli trace ($p_{Ctrl} > \alpha$), with $\alpha = 0.05$ as standard. Notably, this technique produces a specified Type I error rate of 0.05*0.95=4.75%. As such, the expected number of cells falsely taken as gut responsive is $E[n_{positive} = n_{Cells} * \alpha * (1 - \alpha) = n_{Cells} * 0.0475$. Assuming an average of 125,000 cells per fish, this results in an estimated ~6,000 false-positive cells per fish, explaining the large number of detected cells using the vanilla method. As requested, we have now included in the figure panel (now **Supplementary Fig. 7 c,d**) the p-values for the presented cells.
