# Supplementary material for: Whole-brain, all-optical interrogation of neuronal dynamics underlying gut and vascular interoception in zebrafish
Source: Nat Commun. 2026 Aug 27;17:9881. doi: 10.1038/s41467-026-76242-8 (PMC13583002; doi:10.1038/s41467-026-76242-8)
Supplement: Supplementary file 4 [file 41467_2026_76242_MOESM4_ESM.pdf]

Description of Additional Supplementary Files

Title: Supplementary Video 1
Description: Gut stimulus evoked activity across the brain, recorded using light-sheet microscopy in a fish expressing a genetically-encoded calcium indicator in almost all neurons. Trial-average data for one example fish is shown as top, side, and front maximum-intensity projections. Area Postrema, Parabrachial Nucleus, and Nodose ganglion are shown as zoom-ins. Video is 15x sped up. UV stimulus occurs near the start, indicated in the top left.
